# Consequences of Genetic Recombination on Protein Folding Stability

**DOI:** 10.1007/s00239-022-10080-2

**Published:** 2022-12-03

**Authors:** Roberto Del Amparo, Luis Daniel González-Vázquez, Laura Rodríguez-Moure, Ugo Bastolla, Miguel Arenas

**Affiliations:** 1 CINBIO, Universidade de Vigo, 36310 Vigo, Spain; 2 Departamento de Bioquímica, Genética e Inmunología, Universidade de Vigo, 36310 Vigo, Spain; 3 Centre for Molecular Biology Severo Ochoa (CSIC-UAM), 28049 Madrid, Spain; 4 Galicia Sur Health Research Institute (IIS Galicia Sur), 36310 Vigo, Spain

**Keywords:** Recombination, Molecular evolution, Protein evolution, Protein folding stability, Substitution models of protein evolution

## Abstract

Genetic recombination is a common evolutionary mechanism that produces molecular diversity. However, its consequences on protein folding stability have not attracted the same attention as in the case of point mutations. Here, we studied the effects of homologous recombination on the computationally predicted protein folding stability for several protein families, finding less detrimental effects than we previously expected. Although recombination can affect multiple protein sites, we found that the fraction of recombined proteins that are eliminated by negative selection because of insufficient stability is not significantly larger than the corresponding fraction of proteins produced by mutation events. Indeed, although recombination disrupts epistatic interactions, the mean stability of recombinant proteins is not lower than that of their parents. On the other hand, the difference of stability between recombined proteins is amplified with respect to the parents, promoting phenotypic diversity. As a result, at least one third of recombined proteins present stability between those of their parents, and a substantial fraction have higher or lower stability than those of both parents. As expected, we found that parents with similar sequences tend to produce recombined proteins with stability close to that of the parents. Finally, the simulation of protein evolution along the ancestral recombination graph with empirical substitution models commonly used in phylogenetics, which ignore constraints on protein folding stability, showed that recombination favors the decrease of folding stability, supporting the convenience of adopting structurally constrained models when possible for inferences of protein evolutionary histories with recombination.

**Supplementary Information:**

The online version contains supplementary material available at 10.1007/s00239-022-10080-2.

## Introduction

Genetic recombination constitutes a fundamental evolutionary process to acquire molecular diversity (Carroll 2013) and can be observed in multiple organisms, especially viruses (Robertson et al. 1995; Lopes et al. 2014; Perez-Losada et al. 2015; Zhu et al. 2020). Recombination has been associated with the emergence of new organisms (e.g., Ding et al. 2017), increase of viral fitness (e.g., Arenas et al. 2016), intensification of microbial virulence and pathogenesis including therapy and host immunity escape (e.g., Bretscher et al. 2004; Fraser 2005; Perez-Losada et al. 2009, 2015; Shi et al. 2010), or removal of harmful mutations (e.g., Alves et al. 2017). In summary, recombination facilitates evolutionary innovations that would be inaccessible (or too slow) through point mutations alone (Bogarad and Deem 1999).

Concerning the influence of recombination on phylogenetic analyses, molecular fragments involved in recombination events can present different evolutionary histories, leading to incongruent phylogenetic trees, whose combination results in a phylogenetic recombination network usually called as the ancestral recombination graph (ARG) (Griffiths and Marjoram 1997). As a consequence, ignoring recombination affects traditional evolutionary analyses such as phylogenetic tree reconstruction (Schierup and Hein 2000a; Mallo et al. 2016), molecular clock identification (Schierup and Hein 2000b), ancestral sequence reconstruction (Arenas and Posada 2010), and detection of molecular adaptation (Anisimova et al. 2003; Arenas and Posada 2014; Del Amparo et al. 2021), among others (see the reviews Martin et al. 2011; Arenas 2021).

The consequences of recombination can be observed in proteins. For example, recombination is thought to enhance protein adaptation (e.g., Presgraves 2005). The “DNA shuffling” produced by recombination in closely related DNA sequences allows to create novel genes (Stemmer 1994) that can be effective in directed protein evolution (Crameri et al. 1996; Moore et al. 1997; Mutschler et al. 2018).

Although the influence of mutation on protein folding stability was intensively studied (Liberles et al. 2012; Ashenberg et al. 2013; Jiménez-Santos et al. 2018; Strokach et al. 2019; Marcos and Echave 2020), little is known about how protein folding stability is affected by recombination. Recombination events involve an exchange of sequence fragments that can differ by several amino acids, and one could expect that their combination may produce a dramatic loss of protein stability. However, in contrast with this expectation, some experimental studies found that recombination maintains the folding stability of artificial families of cytochrome P450 (Otey et al. 2006; Li et al. 2007), but these studies are difficult to generalize because they exchanged artificially selected stable fragments that belong to related proteins. Using in silico analyses, Xia and Levitt (2002) studied a simplified model of protein folding with two amino acid types and structures on a two-dimensional lattice (HP model), which is amenable to exact computations. They found that neutral evolution under mutation and strong recombination favors the fixation of the prototype sequence of the HP model, which is central to the neutral network and is most robust against mutations.

Here, we extend those previous studies by evaluating the influence of homologous recombination on the computationally predicted folding stability of protein structures evolved under different evolutionary scenarios. We evaluate the influence of the sequence and stability similarity between the recombining (parental) proteins on the stability of the recombined (descendant) proteins. We also compare the effects of recombination and mutation events on the protein folding stability. In particular, we analyze the proportion of recombined and mutated proteins that are eliminated by negative selection on protein folding stability (protein variants that are lost in the population due to selection) under different selection thresholds and for different protein families. In these studies, the parental proteins are stable, because they are evolved adopting a model that implements selection on protein folding stability. Finally, we considered parental proteins evolved under empirical substitution models, which ignore stability constraints but that are traditionally used in phylogenetics (Thorne 2000; Yang 2006; Darriba et al. 2011; Arenas 2015), to find that their protein folding stability decreases rapidly after consecutive recombination events. This suggests that the commonly used empirical substitution models should be replaced by substitution models that consider protein folding stability in order to more realistically model protein evolution along phylogenetic recombination networks.

## Materials and Methods

### Influence of Recombination Events on the Folding Stability of Proteins Simulated Accounting for Structural Constraints

Following previous works (Bastolla et al. 2004; Arenas and Bastolla 2020), we simulated the evolution of five protein families: *D*-alanine *D*-alanine ligase [DDL], Chaperone proteins dnaK [DNAK], Triosephosphate isomerases [TPIS], Tryptophan synthases *α*-chain [TRPA], and Thioredoxins I [TRXB]. All these families are available from the *Pfam* database and include multiple sequences that allow robust evolutionary analyses and a representative protein structure available in the Protein Data Bank (PDB) (Table 1).

**Table 1:** Modeled protein families

Protein family	Gene	Pfam code	Uniprot code	PDB code	Protein length	Sample size	Seq Id	Best-fitting model
*D*-alanine *D*-alanine ligases	DDL	PF07478	DDLB_ECOLI	1IOV	306	42	0.40	LG + I + G
Chaperone proteins dnaK	DNAK	PF00012	DNAK_ECOLI	1DKZ	215	38	0.59	LG + I + G
Triosephosphate isomerases	TPIS	PF00121	TPIS_ECOLI	1TRE	255	32	0.43	LG + I + G
Tryptophan synthases *α* chain	TRPA	PF00290	TRPA_SALTY	1A50	260	25	0.47	LG + G
Thioredoxins I	TRXB	PF00070	TRXB_ECOLI	1TDE	316	28	0.46	LG + I + G

For each protein family, the table shows gene, *Pfam* code, *UniProt* code for a representative protein sequence with a PDB structure, PDB code, sequence length (number of amino acids), sample size (number of sequences), amino acid sequence identity, and the best-fitting empirical substitution model estimated with *ProtTest3*. Note that + I indicates consideration of a proportion of invariable sites and + G indicates consideration of variation of the rate of evolution among sites according to a gamma distribution (Yang 1996)

Firstly, we studied the effect of recombination over stable protein sequences that we simulated imposing constraints on protein folding stability using the evolutionary framework *Prot_evol* (Minning et al. 2013; Arenas et al. 2015). This program simulates protein sequence evolution under structurally constrained substitution (SCS) models of protein evolution (Minning et al. 2013; Arenas et al. 2015). In particular, given a protein structure represented in a PDB file and its associated sequence, the “neutral” version of these SCS models applies random mutations and computationally predicts the folding stability of the mutated protein. This prediction evaluates the difference of free energy *∆G* between the native state and both the unfolded state and the ensemble of compact conformations (misfolded state), which is the peculiarity of our approach. We found that natural protein sequences present clear signals of selection against misfolding (Minning et al. 2013), and we showed that a site-specific substitution model that considers the misfolded state produces higher likelihood, larger stability, and more realistic hydrophobicity values than a similar model that only considers the native and unfolded states (Arenas et al. 2015). The SCS model accepts the mutation if the predicted folding stability is above a threshold proportional to the predicted folding stability of the wild-type (WT) protein sequence whose structure is available in the PDB, i.e., if *∆G* ≤ *t∆G*_*WT*_* ∆G*_*WT*_ is the predicted folding free energy of the PDB sequence, both *∆G* and *∆G*_*WT*_ are predicted using the protein structure of the PDB, and *t* is a user-specified selection parameter. We applied several selective thresholds *t* = 0.99, 0.95, 0.90, 0.75, and 0.50 to explore the influence of this parameter (simulations for which the threshold is not specified were performed with *t* = 0.95).

The program *Prot_evol* simulates a multiple alignment of a user-specified number of protein sequences [without indels to avoid potential biases in the prediction of protein folding stability (Jilani et al. 2022)] evolved under SCS models through independent evolutionary trajectories. The framework *Prot_evol* also provides additional information about the evolutionary process such as the average number of mutation events attempted to reach a substitution (accepted or fixed mutation) event. We simulated 10 independent evolutionary trajectories (star phylogeny), with length 100 stability-constrained substitutions, to obtain 1000 sequences that are predicted to be stable. Next, we randomly sampled 1000 pairs of these sequences [involving from almost identical to 40–50% different, a range that includes commonly observed recombination events (e.g., Mézard et al. 1992; Perez-Losada et al. 2015)] from the multiple sequence alignment and recombined them with breakpoints in all possible positions along the sequences. Note that each homologous recombination involves two parental sequences (recombinant sequences) that produce two descendant sequences (recombined sequences). The sequence identity between parental sequences spanned a broad range, from almost identical to 40–50% different, which includes commonly observed recombination events (e.g., Mézard et al. 1992; Perez-Losada et al. 2015). All in all, for each modeled protein family, we simulated a total of 1000 × *l* recombination events, where *l* is the protein length, which is shown in Table 1 for every protein family. Finally, we estimated with *Prot_evol* the folding free energy of the sequences before and after every mutation and recombination event.

Additionally, we included some illustrative examples of recombination events detected in real data, estimating their consequences on the protein folding stability. In particular, we analyzed recombination in some protein datasets of the highly recombining viruses HIV-1 (Shriner et al. 2004) and HBV (Araujo 2015; Castelhano et al. 2017) (Table S1; Supplementary Material). The datasets were obtained from the Popset database (details in Table S1) and realigned with *MAFFT* (Katoh and Standley 2013). Next, for every dataset, we analyzed the presence of recombination events with the program *RDP4* (Martin et al. 2015). This tool implements several recombination tests and provides the two parent sequences and breakpoints positions for every detected recombination event, which can be used to identify the corresponding recombined sequences. Here, we considered only recombination events statistically supported by at least 2 recombination tests implemented in the program. We also identified the best-fitting protein structure (representative PDB) for each dataset with *Swiss-Model* (Waterhouse et al. 2018) and, finally, we calculated the protein folding stability for the parental and recombined proteins with the methods presented above.

### Influence of Recombination on the Folding Stability of Proteins Simulated Under Empirical Substitution Models Along Phylogenetic Evolutionary Histories

In a second section, we explored the influence of recombination on the folding stability of proteins evolved under empirical substitution models of protein evolution, which are commonly used in phylogenetics (e.g., Gabaldón 2005; Yang 2006; Darriba et al. 2011; Kumar et al. 2018), along ancestral recombination graphs (ARGs). Although these substitution models are well established in the field, they model protein evolution without imposing any stability constraint, so the protein folding stability could be progressively lost. We simulated ARGs with the coalescent modified with recombination (Hudson 1983; Arenas 2019). Note that recombination events in the coalescent are traditionally modeled by recombining two sequences (parental or recombinant sequences) that produce a single descendant (recombined) sequence, because this approach assumes an effective population size much larger than the sample size and, therefore, it is unlikely that both recombined sequences reach the sample (Hudson 1983; Ferretti et al. 2013). For the coalescent simulations, we assumed an effective population size *N* = 1,000, which is a size observed in nature (Waples and England 2011; Lopes et al. 2014). We investigated 6 different levels of population substitution rate (*θ* = 4*Nμl* = 10, 25, 50, 100, 200, and 400) and population recombination rate (*ρ* = 4*Nrl* = 0, 4, 16, 32, 64, and 128), where μ and *r* are the substitution and recombination rates per site per generation, respectively. These parameters produced multiple sequence alignments (MSAs) with typical sequence identities (i.e., 97%, 94%, 89%, 82%, 73%, and 62% for MSAs of the DDL protein family simulated under the *θ* values, respectively, indicated above). The studied population recombination rates are consistent with diverse observations in nature (Stumpf and McVean 2003; Lopes et al. 2014; Castelhano et al. 2017; Arenas 2022). We simulated 100 ARGs for every combination of substitution and recombination rates with the framework *ProteinEvolver* (Arenas et al. 2013). Next, we simulated protein sequence evolution upon the previously simulated evolutionary histories. For each family, we assumed as root sequence the sequence of the representative protein family (with known PDB structure, Table 1) and we evolved that sequence forward in time along the evolutionary history with *ProteinEvolver*. This simulation of protein evolution was performed under the best-fitting empirical substitution model identified with *ProtTest3* (Darriba et al. 2011) for every protein family (Table 1). Note that we assumed neutral evolution in the coalescent evolutionary history and selection in the protein evolution because to our knowledge no current simulation framework implements the simulation of these processes (evolutionary history and molecular evolution) under a same selection process. Thus, this assumption is commonly made in population genetics (see the reviews Yang 2006; Arenas 2012, 2013; Arenas and Posada 2012; Hoban et al. 2012). Finally, the folding free energy of the simulated protein sequences was estimated with the program *DeltaGREM* (Bastolla 2014) based on the protein folding stability model described in Minning, et al. (2013) and also adopted in Arenas et al. (2015). Among other applications, *DeltaGREM* predicts the free energy of every sequence of a MSA with at least one known protein structure accounting for the native, unfolded, and misfolded protein states using the same computation of folding free energy implemented in *ProteinEvolver,* and it was validated through correlations with experimental measures of folding free energy (Bastolla 2014).

## Results

The results are presented in two sections, (i) the influence of recombination events on the folding stability of proteins evolved under stability-constrained substitution (SCS) models, and (ii) the influence of consecutive recombination events on the folding stability of proteins evolved under empirical substitution models that ignore protein stability.

### Influence of Recombination on the Folding Stability of Proteins Evolved Under SCS Models

As indicated in Methods, the applied SCS models accept an evolutionary event if it fulfills *∆G* ≤ *t∆G*_WT_, where *∆G* and *∆G*_WT_ are the folding stability of a descendant protein and a wild-type protein, respectively, and *t* is a user-specified parameter (selection threshold). We start presenting results obtained with *t* = 0.95, and later we investigate the influence of the selection threshold.

First, we evaluated the influence of the position of the recombination breakpoint on the variation of the protein folding stability caused by recombination. We generally found that recombination events occurring at different breakpoint positions do not produce proteins with significantly different folding stability, although recombination breakpoints located at terminal regions showed a lower effect on the variation of the predicted stability (Figures S1–S5; Supplementary Material). Hereafter, we present results for recombination events with breakpoints located in all possible positions and at exactly the middle of the protein.

**Fig. 1:**
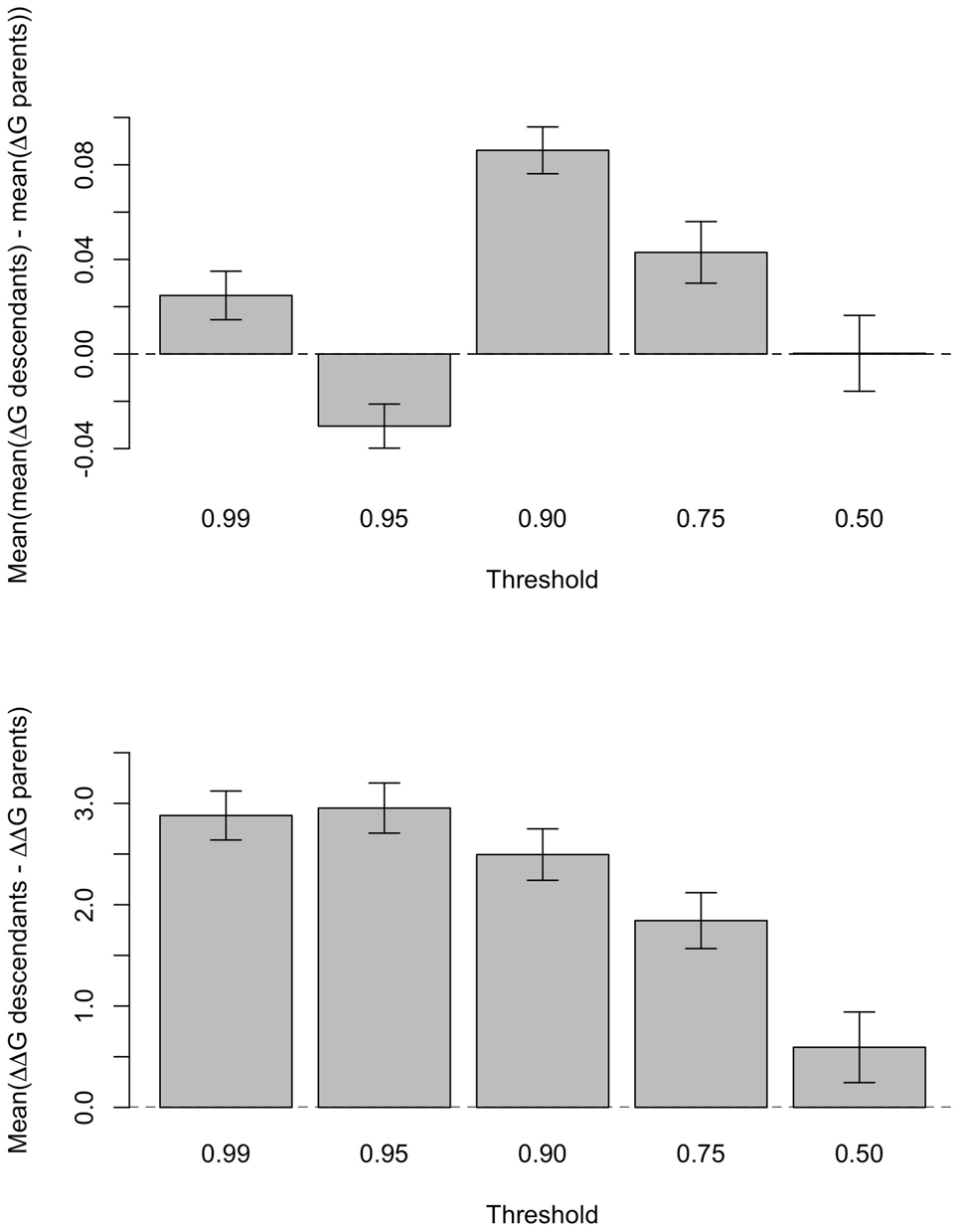
Variation of folding free energy between parental and recombined proteins at varying selection levels. The acceptation of a mutation or recombination event was defined as meeting *∆Gs* ≤ *t∆Gr*, where *∆Gs* is the folding stability of the tested protein (i.e., generated by a mutation or recombination event), *∆Gr* is the folding stability of the real protein (Table 1), and *t* is a user-specified selection threshold. We recombined stable proteins according to this criterion, and considered all recombined proteins, either stable or unstable. The plots show the difference in folding free energies between parent and recombined (descendant) protein sequences (*y*-axis) as a function of the selection threshold (*x-*axis). Plot above: difference of mean. The mean of the folding free energies of the descendants is only slightly different from the mean of the parents (note the small scale of the *y*-axis). Plot below: difference of differences. The difference of the folding free energies of the descendants is much larger than the same difference of the parents. Results based on simulations of the DDL protein family. Error bars represent the 95% confidence interval of the mean, assuming that different protein pairs are independent

Comparing predicted protein folding free energy before and after recombination, we found a high correlation between the mean folding free energy of parental and recombined sequences (e.g., for DDL, correlation coefficient = 0.989 with *p* value < 2.2e^−16^; Figs, S6–S10; Supplementary Material). Therefore, highly stable parental recombinant proteins tend to produce highly stable recombined proteins and the opposite. On the other hand, the difference in stability ($\Delta \Delta G$) between the recombined proteins is almost uncorrelated with the difference in stability between their parents, thus parental proteins with similar folding free energy can produce descendants with rather different folding free energy, and vice versa (Figs. S11–S15; Supplementary Material). In particular, we found that the mean protein folding stability of the recombined proteins is almost identical to the one of the parental proteins (Fig. 1; upper plot). There is a weak tendency that the folding free energy increases and the stability decreases after recombination events, but this effect is small (mean differences of order of hundredths of energy units). In contrast, the difference in free energy between the two descendant proteins is much larger than the same difference for the parental proteins, with differences of almost 3 energy units, especially at high selection thresholds where highly stable proteins recombine (Fig. 1; below). This phenomenon promotes diversity even when the parent proteins have similar properties.

**Fig. 2:**
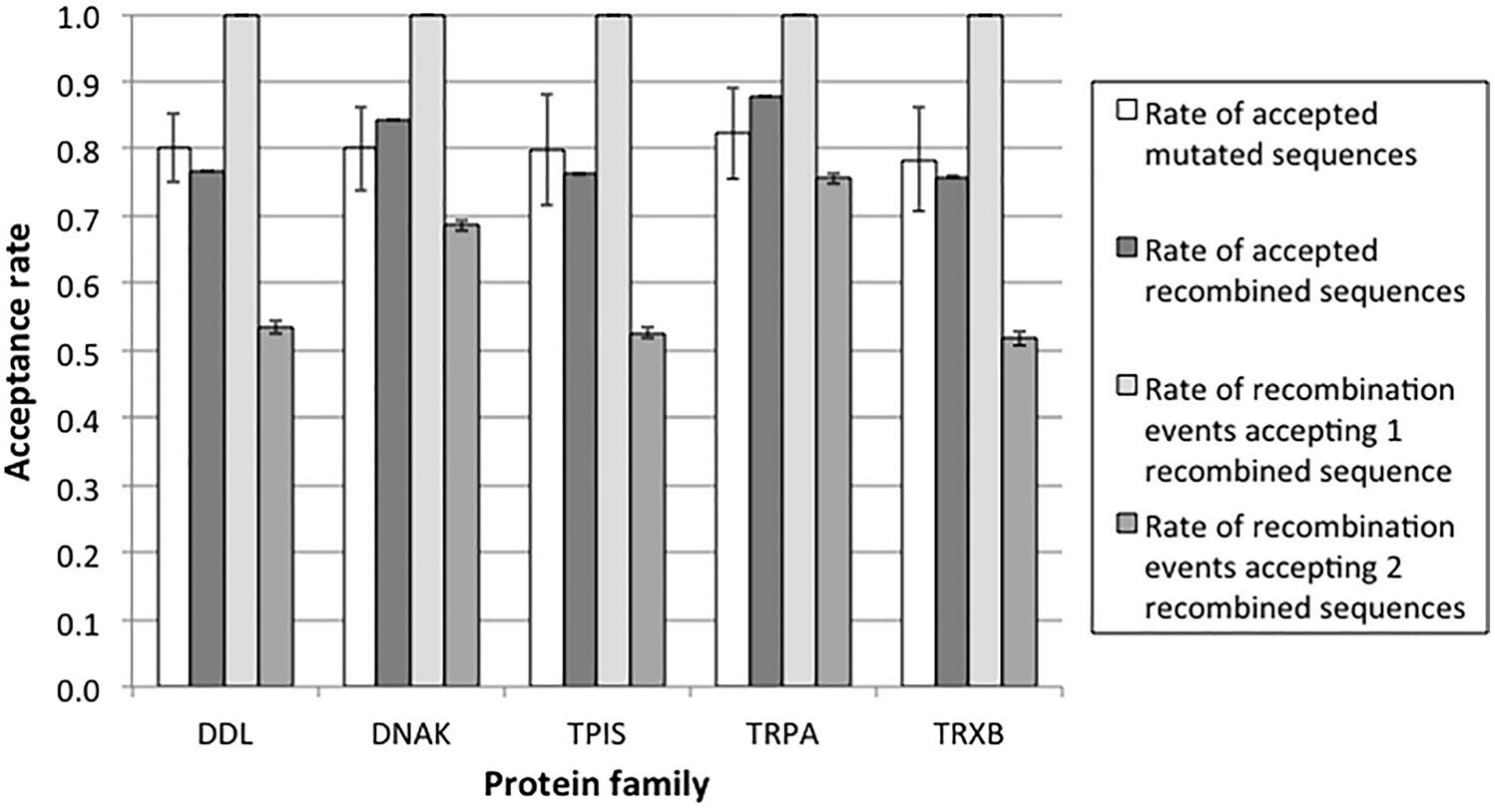
Acceptance rates of mutated and recombined sequences in several protein families. The acceptation of a mutation or recombination event was defined as meeting *∆Gs* ≤ *t∆Gr*, where *∆Gs* is the folding stability of the tested protein (i.e., generated by a mutation or recombination event), *∆Gr* is the folding stability of the real protein (Table 1), and *t* is a user-specified threshold. In this figure, the threshold is 0.95. The figure shows the acceptance rates of mutated sequences and recombined sequences, as well as the rates of recombination events accepting only one recombined sequence and both recombined sequences. Error bars correspond to the standard error of the mean of the respective mutation or recombination events. Results for the same analysis but focused on recombination events with breakpoints occurring only in the middle position of sequences are shown in Fig. S16

**Fig. 3:**
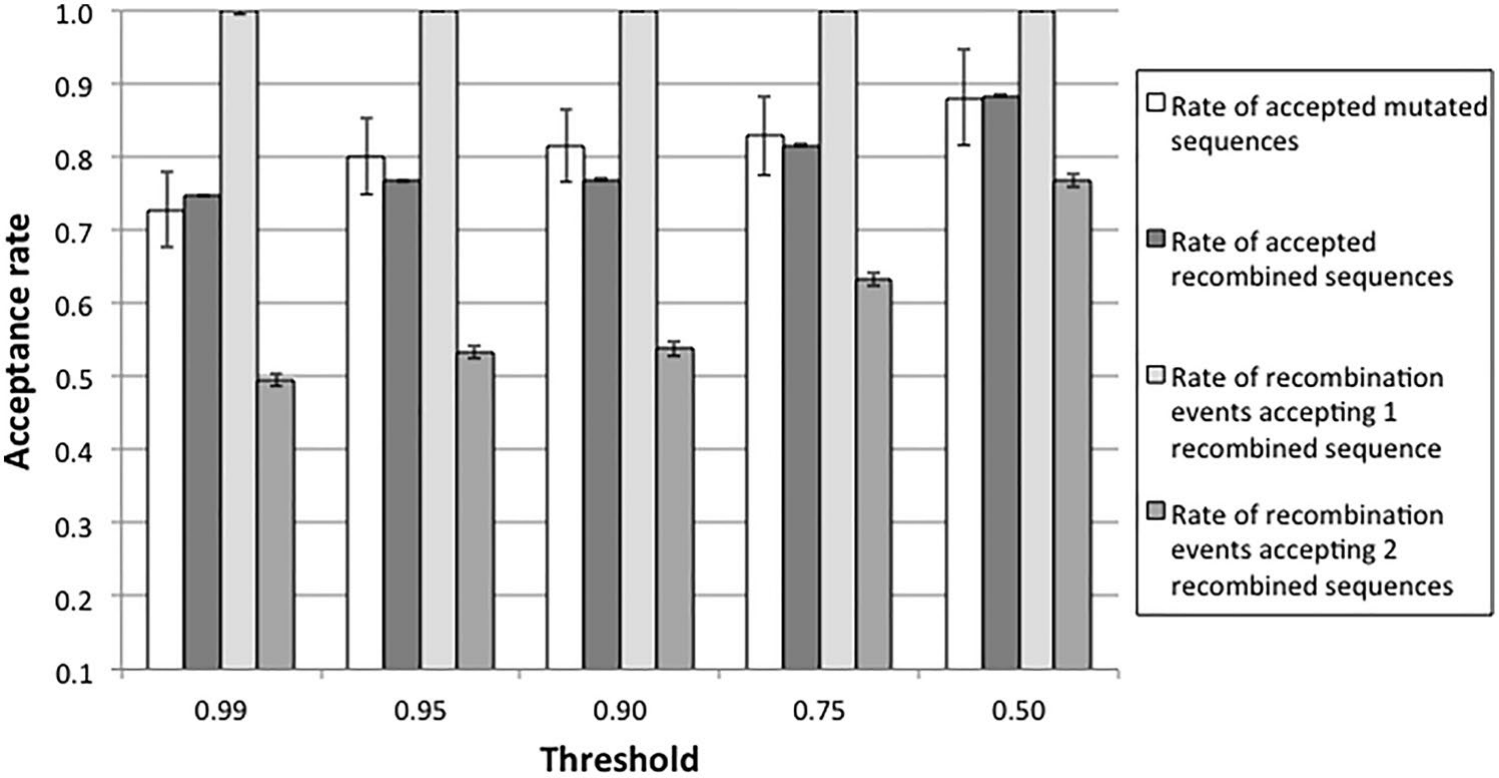
Acceptance rates of protein sequences derived from mutation and recombination events at variable selection levels. The acceptation of a mutation or recombination event was defined as meeting *∆Gs* ≤ *t∆Gr*, where *∆Gs* is the folding stability of the tested protein (i.e., generated by a mutation or recombination event), *∆Gr* is the folding stability of the real protein (Table 1), and *t* is a user-specified threshold. The figure shows the acceptance rates of mutated sequences and recombined sequences, as well as the rates of recombination events accepting only one recombined sequence and both recombined sequences. Results based on simulations of the DDL protein family. Error bars correspond to the standard error of the mean of the respective mutation or recombination events

Next, we compared the fraction of sequences produced by mutation events and by recombination events that are more stable than the neutral threshold and that would be maintained by purifying selection. In Figs. 2 and S16 (Supplementary Material), we represent the acceptance rate as the fraction of the sequences produced by mutation and recombination events, respectively, that have stability above the threshold. The results indicate that the two acceptance rates are similar. Since homologous recombination produces two new sequences, we also evaluated the rate of recombination events that produce one or two recombined sequences above threshold (stable). We found that almost all the recombination events produce at least one stable descendant, while recombination events where the two descendants are stable are less frequent but still above 50% (Figs. 2 and S16). These results indicate that the consequences of recombination and mutation on the protein folding stability are not much different. We then explored the effect of the selection threshold *t*, finding that the qualitative results described above are rather robust under variation of this parameter (Fig. 3). As expected, decreasing the selection parameter results in accepting more sequences for both mutation and recombination, whose acceptance rates are still similar (Fig. 3). Recombination events that produce at least one accepted descendant protein were more frequent than accepted mutation events, while recombination events that produce two accepted descendant proteins, which involve a more restrictive criterion, were less frequent than accepted mutation events (Figs. S17 and S18; Supplementary Material).

**Fig. 4:**
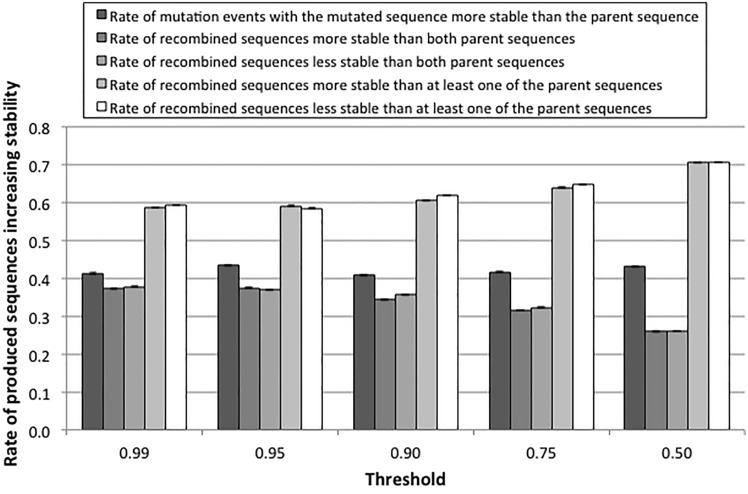
Rates of accepted mutated and recombined sequences that are more stable or unstable than their parent sequences at diverse selection levels. The figure shows the rate of mutated sequences more stable than their parent sequences and the rates of recombined (descendant) sequences that are more stable or unstable than both or one of the parental sequences. Results based on simulations of the DDL protein family. Error bars indicate standard error of the mean of the corresponding mutation and recombination events. This evaluation considers recombination events with breakpoints located in all the positions. Results for the same analysis but focused on recombination events with breakpoints occurring only in the middle position of sequences are shown in Fig. S20

For all the protein families, we also compared the fraction of proteins produced through mutation and recombination that are more stable or unstable than their parents (Fig. S19; Supplementary Material) and as a function of the selection threshold (Figs. 4 and S20; Supplementary Material). We found that the fraction of recombined proteins that are more stable than both parents is almost the same as the fraction of mutated proteins more stable than their parent, especially if the selection threshold is high (38% compared with 42% for a threshold of 0.95). The fraction of recombined proteins that are more stable than at least one of the parents is much higher than for mutation (at least 60%). This indicates that it is relatively easy to increase (or decrease) protein folding stability through recombination. The selection level influences the fraction of mutated sequences that increase their stability. Decreasing the selection level decreases the fraction of recombined proteins that are more stable or unstable than both parents, and increases the fraction of recombined proteins that have intermediate stability between the parental sequences (Figs. 4 and S20). This fraction is less than 50% for low stability threshold of 0.5, and it decreases if the selection threshold increases, while the fraction of proteins more stable or unstable than both parents increases (Fig. 4). This suggests that the stronger is the selection the larger is the phenotypic diversity produced by recombination.

**Fig. 5:**
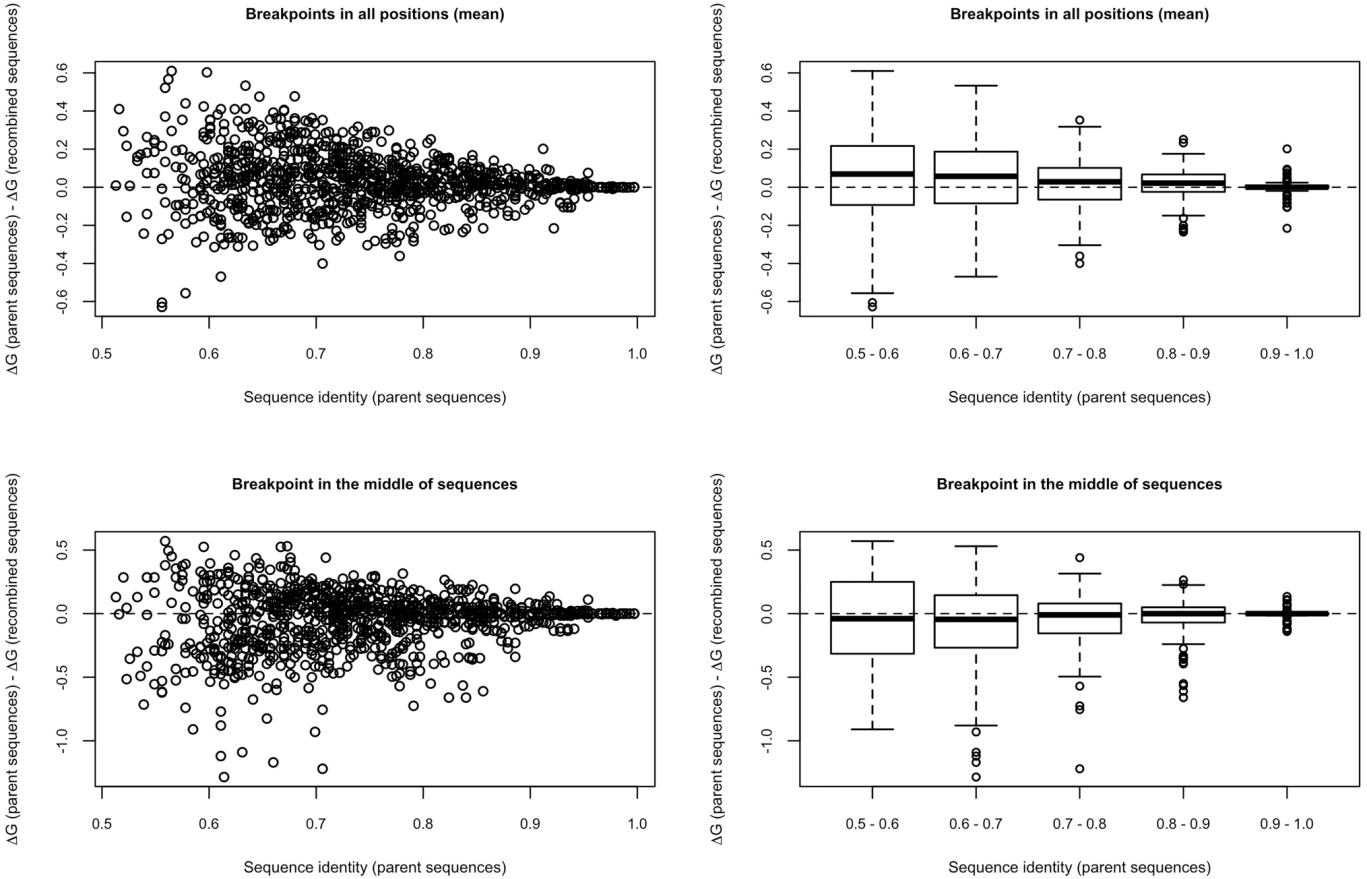
Influence of sequence identity between parental sequences on the folding free energy caused by recombination in the protein family DDL. The figure shows the folding free energy variation produced by recombination (∆∆G) between recombinant (parental) and recombined (descendant) sequences. Negative values mean that the two sequences before recombining are more stable (mean) than the two sequences after recombining (mean), and the opposite for positive values, as a function of the sequence identity (shown on the right by intervals) between the parental sequences. Results based on a selection threshold of 0.95. The above plots refer to recombination events occurring in all the breakpoint positions (mean) and plots below refer to recombination events with breakpoint position only located in the middle of the sequences. Results for other protein families are shown in Figs. S21–24

Next, we compared the mean stability of parental and descendant proteins involved in recombination events as a function of the amino acid sequence identity between the parental proteins. Not surprisingly, we found that recombination between similar proteins with high sequence identity generally leads to descendant proteins with folding stability similar to those of the parents (Figs. 5 and S21–S24; Supplementary Material). Nevertheless, when the parental proteins present different sequences, the stability of the descendants is more heterogeneous, favoring the generation of phenotypic diversity. We also explored the consequences of recombination events observed in some illustrative datasets from viruses (Table S1) on the folding stability. In agreement with the findings from simulated data, we found minor effects of these recombination events on the predicted protein stability (Fig. S25; Supplementary material). In particular, the recombined sequences presented stability close to the stability of the corresponding parents. In addition, also in agreement with the results from simulated data, we found that recombination events between proteins with lower sequence identity can produce larger changes on the protein folding stability (Fig. S26; Supplementary material).

### Influence of Recombination on the Folding Stability of Proteins Evolved Under Empirical Substitution Models Along Phylogenetic Evolutionary Histories

**Fig. 6:**
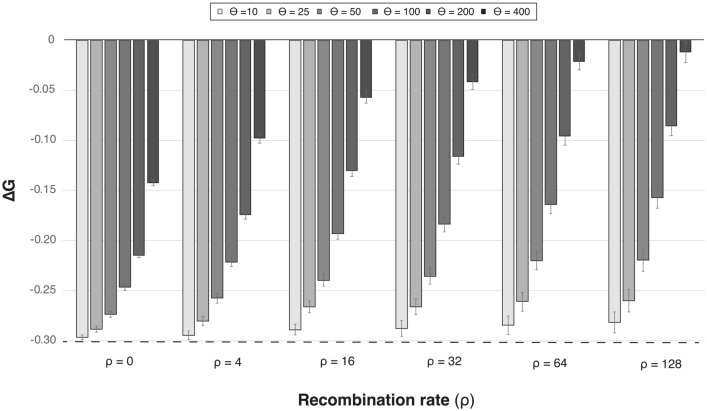
Folding free energy of DDL proteins simulated upon coalescent trees with diverse combinations of population substitution and recombination rates. Folding free energy (∆G) of proteins simulated upon coalescent trees previously simulated under a variety of combinations of population substitution rate (*θ*) and population recombination rate (*ρ*) and where the protein sequences evolved under the best-fitting empirical substitution model (Table 1). The dashed line corresponds to the ∆G of the extant protein structure of the protein family (Table 1). Error bars represent the 95% confidence interval among the mean of computer simulations. Results for other protein families are shown in Fig. S28

In this section, we studied the influence on protein folding stability of recombination events that are modeled without any constraint on stability, i.e., applying empirical substitution models. Before exploring recombination, we investigated the folding stability of proteins modeled under this type of substitution models. We found that protein sequences simulated under empirical substitution models are unrealistically more unstable than proteins simulated with substitution models that consider the protein structure (Fig. S27; Supplementary material), confirming previous results (Arenas et al. 2013; Bordner and Mittelmann 2013). Indeed, increasing the substitution rate, which produces longer branches and thus more substitutions are incorporated, amplified the instability of the simulated proteins at any level of simulated recombination rate (Figs. 6 and S28; Supplementary Material). In addition, protein sequences simulated under empirical substitution models along an ARG based on a large recombination rate showed a further decrease of folding stability (Figs. 6 and S28). The strength of this bias caused by recombination depended on the substitution rate, producing a stronger decrease of stability when protein evolution is simulated under a large substitution rate. These results underscore the importance of taking into account protein folding stability in simulations of protein evolution in order to avoid proteins with unrealistic physicochemical properties.

From the perspective of every recombination event present in the simulated ARG, we found that recombination events involving recombining (parental) proteins with large sequence identity produced recombined (descendant) proteins with folding stability similar to that of the parental proteins, while dissimilar recombining proteins produced proteins with a more broadly distributed folding free energy compared to that of the recombining proteins (Figs. S29–S33; Supplementary Material). In particular, we found that a large fraction of recombination events produce proteins with folding free energy in between those of the corresponding recombinant sequences. This fraction ranges from 48 to 80%, depending on the substitution rate (Figs. S29–S33 and Table S2; Supplementary Material). In contrast, this fraction was smaller (around one third) for proteins evolved with stringent stability constraints (selection threshold *t* = 0.99), but it increased to approximately 50% for more tolerant selection on folding stability (*t* = 0.5, see Fig. 4), which is consistent with the results in the absence of selection on folding stability. A smaller fraction of recombined sequences increased or decreased, in similar proportion, folding stability with respect to their corresponding parental sequences. This is also consistent with results based on SCS models (Fig. 1, below), where the difference between the stability variation of descendants and parents tends to decrease with the selection threshold. Therefore, the findings obtained in the absence of stability overall agree with the trends from the selection threshold *t* that were presented in the previous section (evolving sequences under SCS models; Figs. 5 and S21–S24). However, recombination applied without stability constraints strongly hinders protein stability, especially when the recombining proteins are distant homologous, making protein evolution under empirical models together with recombination even less realistic under the point of view of protein stability.

## Discussion

Recombination is a common evolutionary force that produces molecular diversity (Carroll 2013) and must be taken into account in phylogenetic inferences (Schierup and Hein 2000a, b; Anisimova et al. 2003; Mallo et al. 2016). However, the consequences of recombination on the protein folding stability are still little investigated. A few experimental studies showed that recombination can maintain protein folding stability (Otey et al. 2006; Li et al. 2007) but those studies involved selected recombination events among very similar proteins. At the beginning of this study, we hypothesized a strong loss of stability in recombination events. Nevertheless, the results showed that a large fraction of recombination events produce proteins with folding stability in between the folding stability of the corresponding parental proteins, especially if the parental proteins are similar in terms of sequence identity and folding stability. More importantly, we found that the probability that a protein produced by recombination is eliminated by purifying selection because of insufficient folding stability is similar to the same probability for proteins produced through a point mutation. These results agree with the previously reported experimental observations (Otey et al. 2006; Li et al. 2007), although more experimental evidence is needed for a thorough comparison between recombination and point mutation. Our study only considers recombination events between proteins that belong to the same family and fold into a common protein structure. Here, we did not explore the consequences for protein folding stability of recombination events between proteins that belong to different protein families or match with different protein structures, because recombination events usually occur in nature between similar sequences (e.g., Mézard et al. 1992; Perez-Losada et al. 2015), and because, to our knowledge, all currently available SCS models impose stability constraints based on only one protein structure (Liberles et al. 2012). Indeed, protein evolution often includes the recruitment of domains through diverse evolutionary processes (i.e., horizontal gene transfer, retrotransposition and genetic recombination) that involve exchange of genetic material (Basu et al. 2009; Yang and Bourne 2009; Bagowski et al. 2010; Dohmen et al. 2020; Aziz and Caetano-Anollés 2021). These evolutionary events that involve combining genetic material from unrelated parents are thought to play an important role for creating phenotypic novelty at the structural and functional level, but they are outside the scope of the present study.

We found a striking similarity between the effects of recombination and point mutation on protein folding stability. This result is at first sight surprising, because recombined proteins present several amino acid differences with respect to their parents as opposed to the single amino acid change of point mutants, and thus one may naively expect that that it is more likely that purifying selection eliminates them. Furthermore, it is surprising that the mean stability of recombined proteins is similar to that of their parents, because recombination disrupts epistatic interactions (Otto and Feldman 1997; McLeod and Gandon 2022).

In real proteins, short-range contacts are not crucial for folding stability because they are shared by both the native structure and incorrectly folded conformations and tend to be destabilized through negative design. Indeed, the stabilizing energy of a contact tends to increase with the sequence separation along the chain (Minning et al. 2013). For this reason, we might expect that most of native interactions that are disrupted by recombination are important for protein stability. However, protein stability has a twofold nature, (1) one-body stability of a given amino acid at a given position (i.e., hydrophobic amino acids at buried positions) and (2) two-body stability conferred by specific interactions such as salt bridges or hydrogen bonds that involve side chains. Our previous studies of site-specific amino acid frequencies indicated that the body contribution is the most relevant (Minning et al. 2013), and this could be a reason for the observed mild consequences of recombination on protein folding stability.

In our opinion, the high similarity in the mean stability of the descendants and parents supports the approaches that model evolution with selection on protein stability through site-specific but site-independent substitution processes, such as for instance the mean-field SCS model of protein evolution (Bastolla et al. 2006; Minning et al. 2013; Arenas et al. 2015). These approaches consider independent substitution processes at each site subjected to a global constraint on protein folding stability, an approximation that reduces the influence of epistatic interactions. Note that recombination switches amino acids that were previously tested by natural selection, which also holds in our numerical experiments where the parents are stable by construction. If we separate the contribution of each amino acid to stability into single-site contributions (such as hydrophobic amino acids at buried position, or secondary structure propensity) and pairwise (epistatic) contributions, we expect that the mean of the single-site contributions does not change between the parents and the descendant proteins, but the pairwise contributions to stability should decrease after recombination. However, this decrease of the mean stability is very weak or absent in our numerical experiments (Fig. 1A) despite our use of a pairwise energy function based on contact interactions, which supports the idea that the independent sites approximation used in our mean-field model is acceptably good.

On the other hand, recombination creates pairs of proteins whose stabilities differ more than those of their parents (Fig. 1B), supporting the view that recombination amplifies phenotypic diversity. An interpretation of this effect, given the previous result that suggests that epistatic interactions are not very different between parents and recombined proteins (i.e., the studied recombination did not reflect dependency between sites), is that different proteins that evolved under stability constraints tend to have similar stability, but this can be differently distributed across the protein sequence. For example, if the *N* terminal region of protein A is more stable than the *N* terminal region of protein B but the opposite happens for the *C* terminal region, their recombination, neglecting epistatic interactions, will tend to produce proteins whose stabilities are outside the range of the stability of the parents. Interestingly, this difference between parents and daughters decreases with the selection threshold, being smaller for proteins evolved under less stringent selection (Fig. 2B).

This selection is completely absent for protein sequences that evolve under empirical substitution models, which are unaware of protein folding stability. Nevertheless, these models are commonly used in phylogenetics [i.e., most currently available computer programs for protein phylogenetic analysis implement only empirical substitution models (Arenas 2015; Bouckaert 2020; Darriba et al. 2020; Minh et al. 2021)]. As expected, we confirmed that these models produce unrealistically unstable proteins, which become even more unstable after recombination events especially in proteins that evolved under large substitution rates. In agreement with previous studies but focused on mutation (e.g., Liberles et al. 2012; Wilke 2012; Bordner and Mittelmann 2013; Larson et al. 2020), these findings recommend considering the modeling of substitution and recombination processes accounting for protein folding stability.

In this study, we adopted a simplified model of evolution that considers selection on protein folding stability assuming that the mutation does not change the protein structure. In our opinion, the strongest selection operates on the protein function and, through it, on the protein structure. This opinion is supported by the observation that the strongest signatures of both negative and positive selection appear in the TM score that quantifies the evolutionary divergence of the backbone traces of protein structures. The second strongest signatures of negative and positive selection appear in the contact overlap, which is affected by both structure and protein folding stability changes. The weakest selection seems to operate on protein sequences. Thus, sequence changes without divergence of protein structures would only affect folding stability and could be tolerated more easily than structural changes (Pascual-Garcia, et al. 2019). Therefore, it would be useful to improve the selection model in order to also take into account possible changes of the protein structure and its functional dynamics.

## Supplementary Information

Below is the link to the electronic supplementary material.

Supplementary file1 (PDF 4900 KB)

## Data Availability

The real data are available from databases (accession codes are included in Tables 1 and S1). The simulated data are available at Zenodo repository from the URL https://doi.org/10.5281/zenodo.6814286.
