# Supplementary material for: Consequences of Genetic Recombination on Protein Folding Stability
Source: J Mol Evol. 2022 Dec 3;91(1):33–45. doi: 10.1007/s00239-022-10080-2 (PMC9849154; doi:10.1007/s00239-022-10080-2)
Supplement: Supplementary file 1 — Supplementary file1 (PDF 4900 KB) [file 239_2022_10080_MOESM1_ESM.pdf]

Supplementary Material

Consequences of genetic recombination on protein folding stability

The supplementary material includes Figures S1-S33, Tables S1-S2 and references cited in the supplementary material.

Figure S1. Folding free energy variation caused by recombination events at every breakpoint position in the protein family DDL. For every breakpoint position, the figure shows boxplots with the variation of free energy caused by recombination (difference between folding free energy of parental and descendant proteins). Note that the site boxplot distributions overlap, indicating an overall lack of statistical differences. However, breakpoints located at extreme regions of the sequences showed a trend of reducing effects of recombination on the folding free energy.

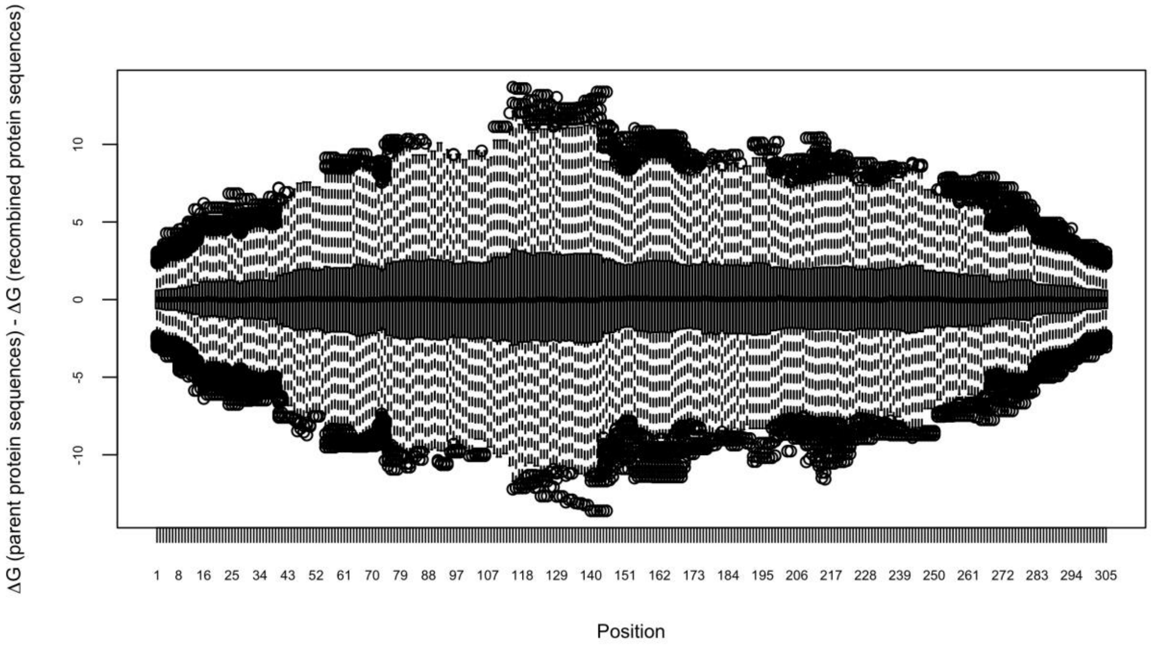

Figure S2. Folding free energy variation caused by recombination events at every breakpoint position in the protein family DNAK. For every breakpoint position, the figure shows boxplots with the variation of free energy caused by recombination (difference between folding free energy of parental and descendant proteins). Note that the site boxplot distributions overlap, indicating an overall lack of statistical differences. However, breakpoints located at extreme regions of the sequences showed a trend of reducing effects of recombination on the folding free energy.

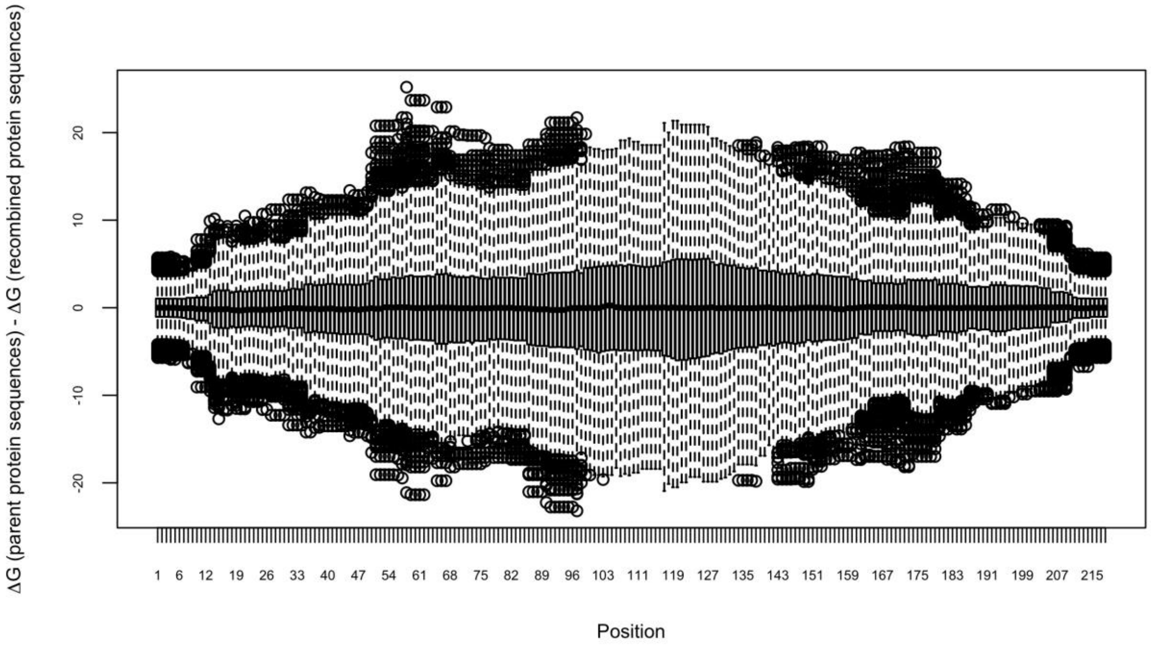

Figure S3. Folding free energy variation caused by recombination events at every breakpoint position in the protein family TPIS. For every breakpoint position, the figure shows boxplots with the variation of free energy caused by recombination (difference between folding free energy of parental and descendant proteins). Note that the site boxplot distributions overlap, indicating an overall lack of statistical differences. However, breakpoints located at extreme regions of the sequences showed a trend of reducing effects of recombination on the folding free energy.

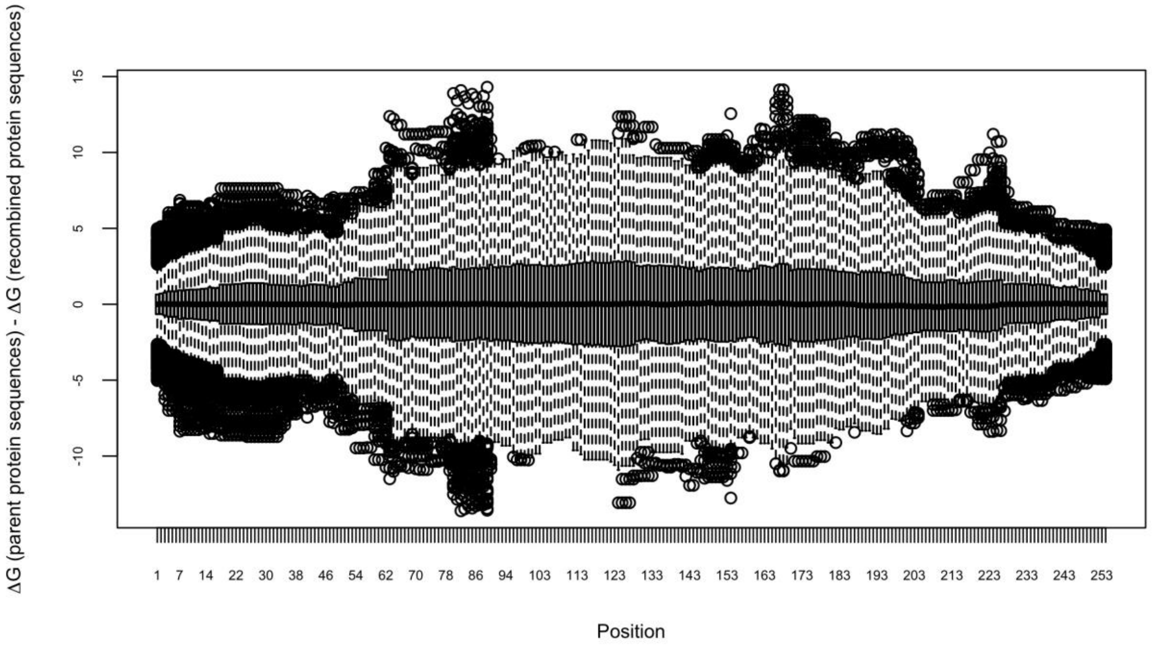

Figure S4. Folding free energy variation caused by recombination events at every breakpoint position in the protein family TRPA. For every breakpoint position, the figure shows boxplots with the variation of free energy caused by recombination (difference between folding free energy of parental and descendant proteins). Note that the site boxplot distributions overlap, indicating an overall lack of statistical differences. However, breakpoints located at extreme regions of the sequences showed a trend of reducing effects of recombination on the folding free energy.

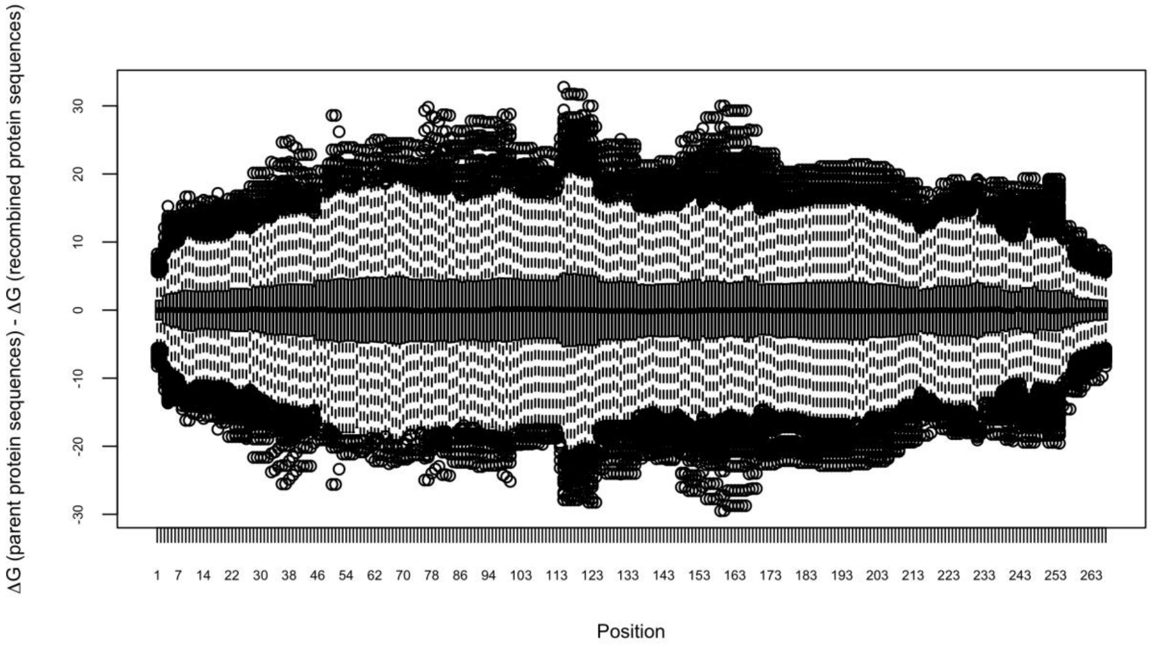

Figure S5. Folding free energy variation caused by recombination events at every breakpoint position in the protein family TRXB. For every breakpoint position, the figure shows boxplots with the variation of free energy caused by recombination (difference between folding free energy of parental and descendant proteins). Note that the site boxplot distributions overlap, indicating an overall lack of statistical differences. However, breakpoints located at extreme regions of the sequences showed a trend of reducing effects of recombination on the folding free energy.

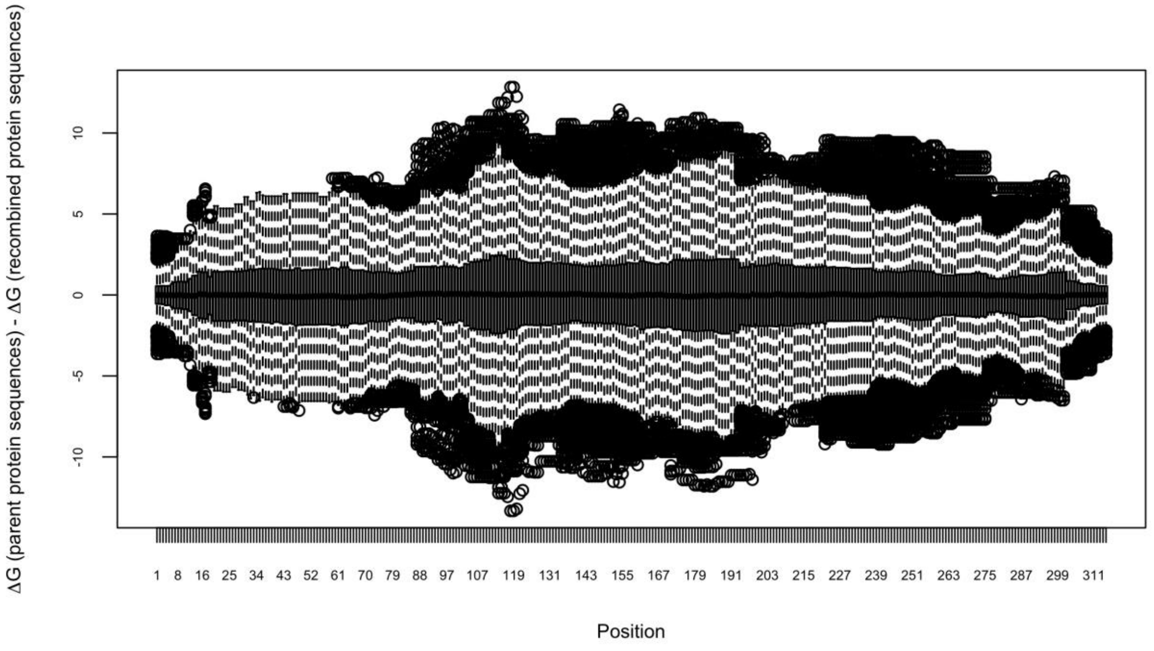

Figure S6. Folding free energy of recombined protein sequences as a function of folding free energy of parental protein sequences for the protein family DDL. The plots show the mean of folding free energy of parent protein sequences (*y axis*) as a function of the mean of folding free energy of recombined protein sequences (*x axis*) in a total of 1,000 recombination events. Above plot considers recombination events at every breakpoint position (mean); correlation coefficient = 0.989, p value < $2.2e^{-16}$. Below plot, relationship considering recombination events with breakpoint position located in the middle of the sequences (correlation coefficient = 0.984, p value < $2.2e^{-16}$).

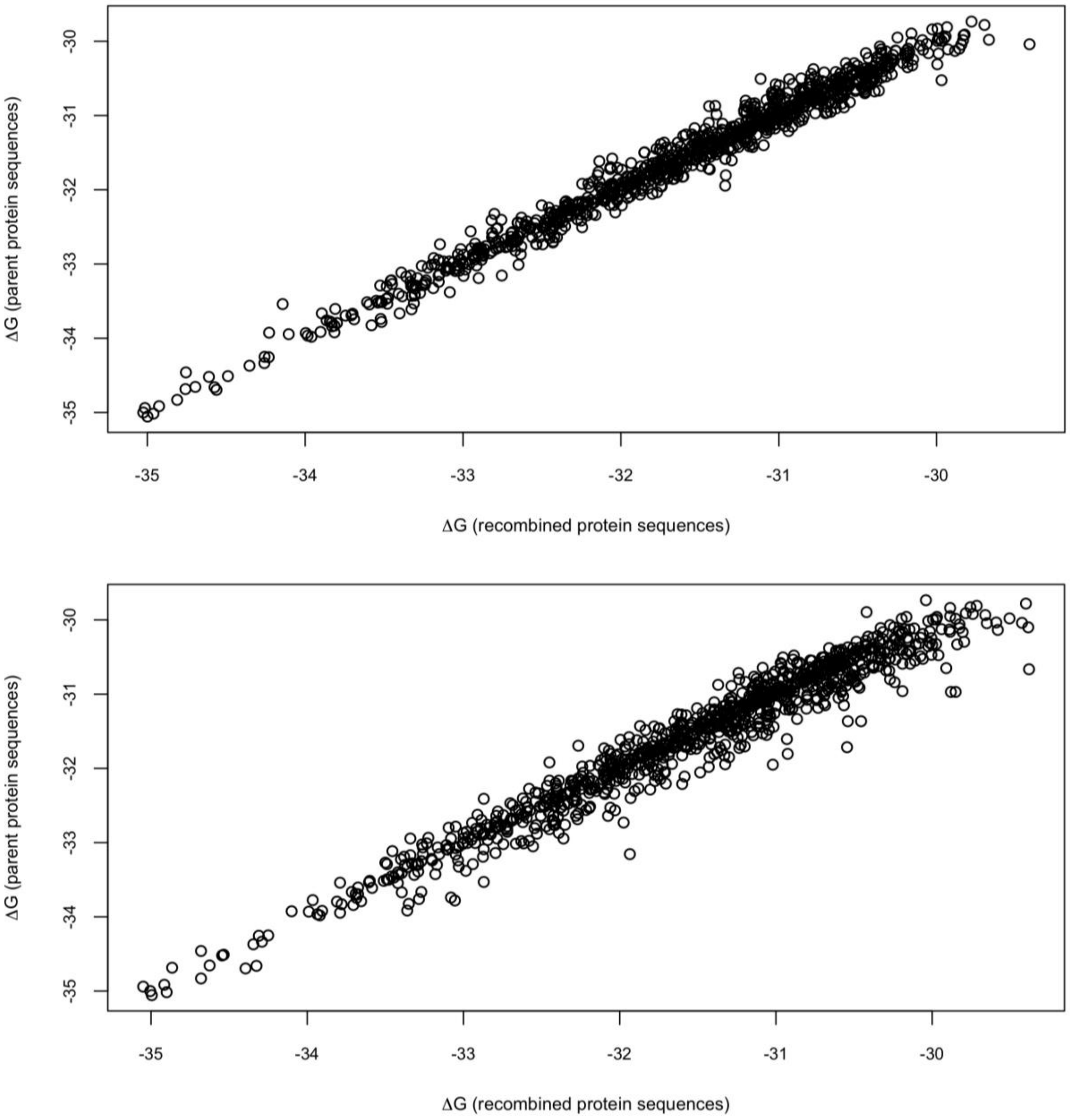

Figure S7. Folding free energy of recombined protein sequences as a function of folding free energy of parental protein sequences for the protein family DNAK. The plots show the mean of folding free energy of parent protein sequences (*y axis*) as a function of the mean of folding free energy of recombined protein sequences (*x axis*) in a total of 1,000 recombination events. Above plot considers recombination events at every breakpoint position (mean); correlation coefficient = 0.988, p value < $2.2e^{-16}$. Below plot, relationship considering recombination events with breakpoint position located in the middle of the sequences (correlation coefficient = 0.954, p value < $2.2e^{-16}$).

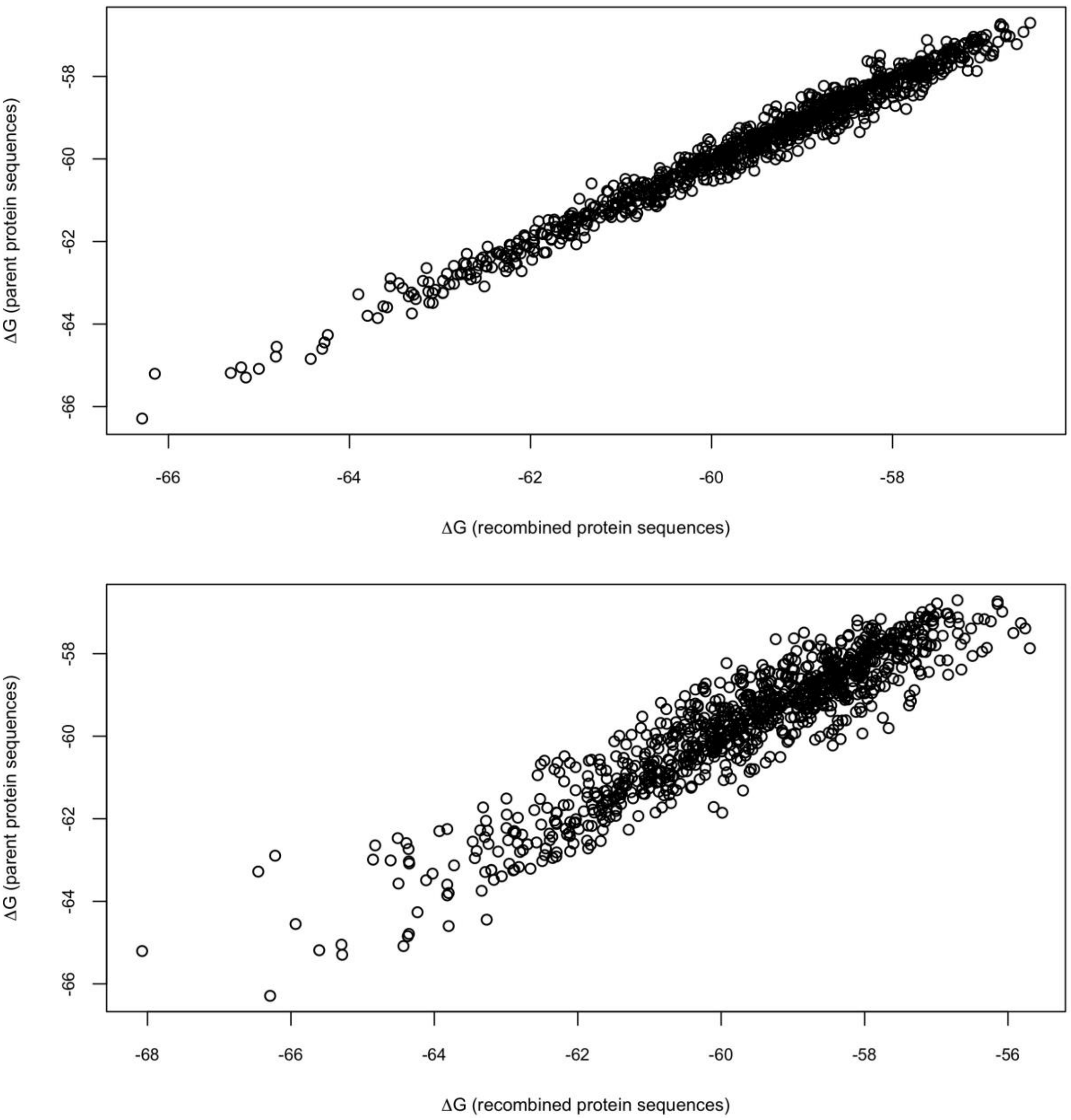

Figure S8. Folding free energy of recombined protein sequences as a function of folding free energy of parental protein sequences for the protein family TPIS. The plots show the mean of folding free energy of parent protein sequences (*y axis*) as a function of the mean of folding free energy of recombined protein sequences (*x axis*) in a total of 1,000 recombination events. Above plot considers recombination events at every breakpoint position (mean); correlation coefficient = 0.977, p value < $2.2e^{-16}$. Below plot, relationship considering recombination events with breakpoint position located in the middle of the sequences (correlation coefficient = 0.908, p value < $2.2e^{-16}$).

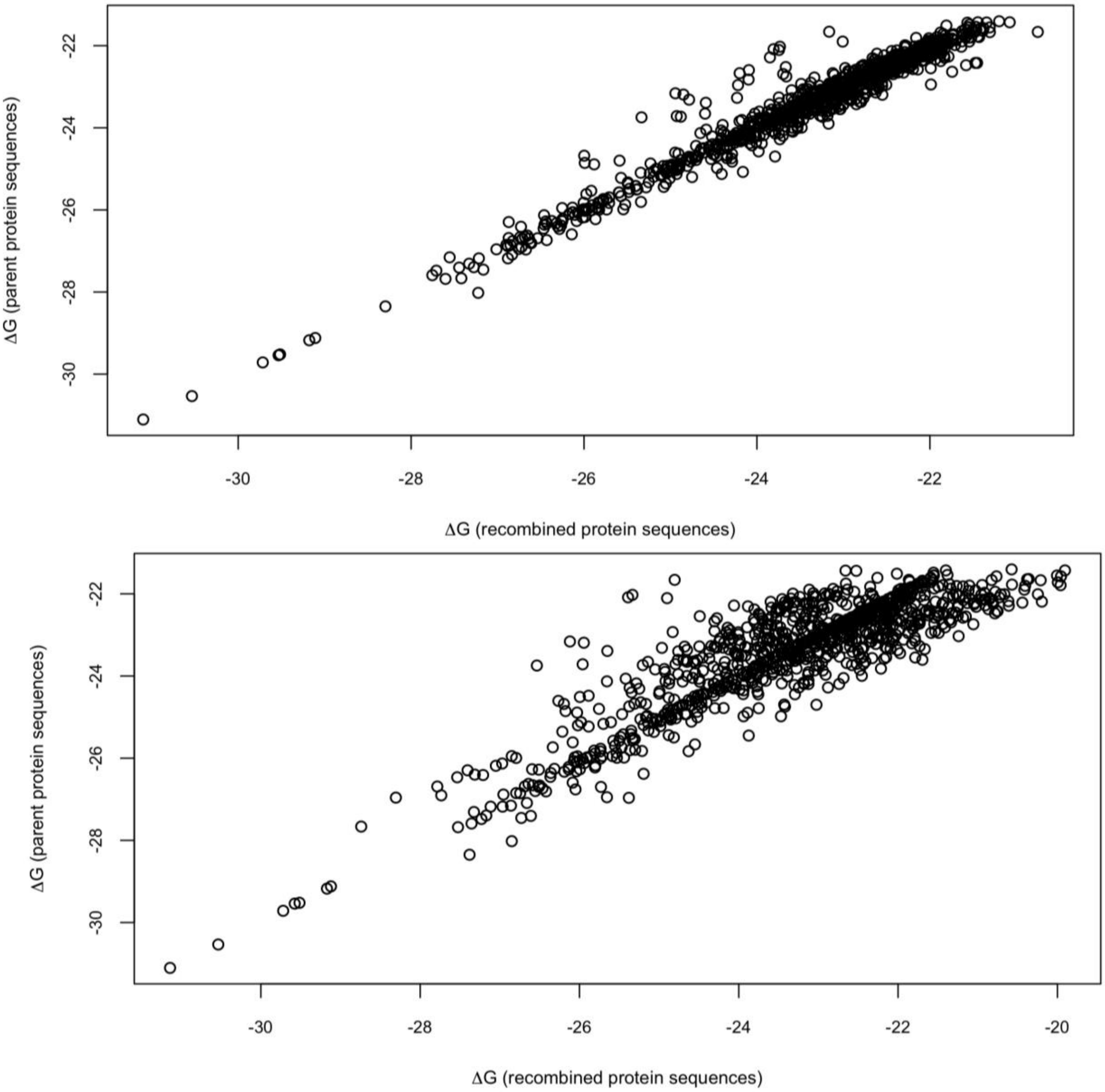

Figure S9. Folding free energy of recombined protein sequences as a function of folding free energy of parental protein sequences for the protein family TRPA. The plots show the mean of folding free energy of parent protein sequences (*y axis*) as a function of the mean of folding free energy of recombined protein sequences (*x axis*) in a total of 1,000 recombination events. Above plot considers recombination events at every breakpoint position (mean); correlation coefficient = 0.985, p value < $2.2e^{-16}$. Below plot, relationship considering recombination events with breakpoint position located in the middle of the sequences (correlation coefficient = 0.986, p value < $2.2e^{-16}$).

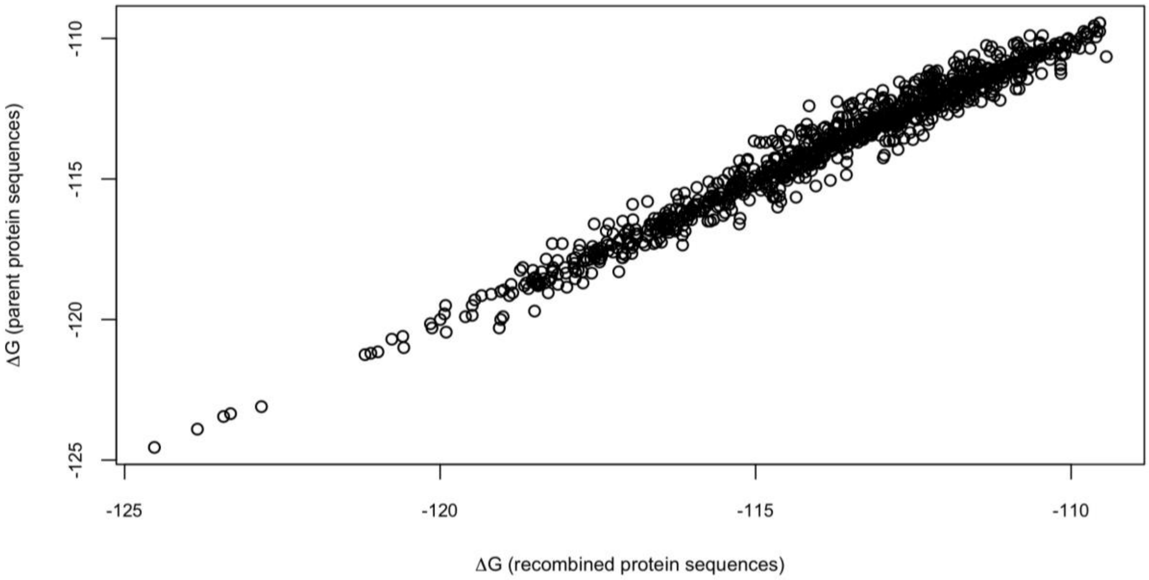

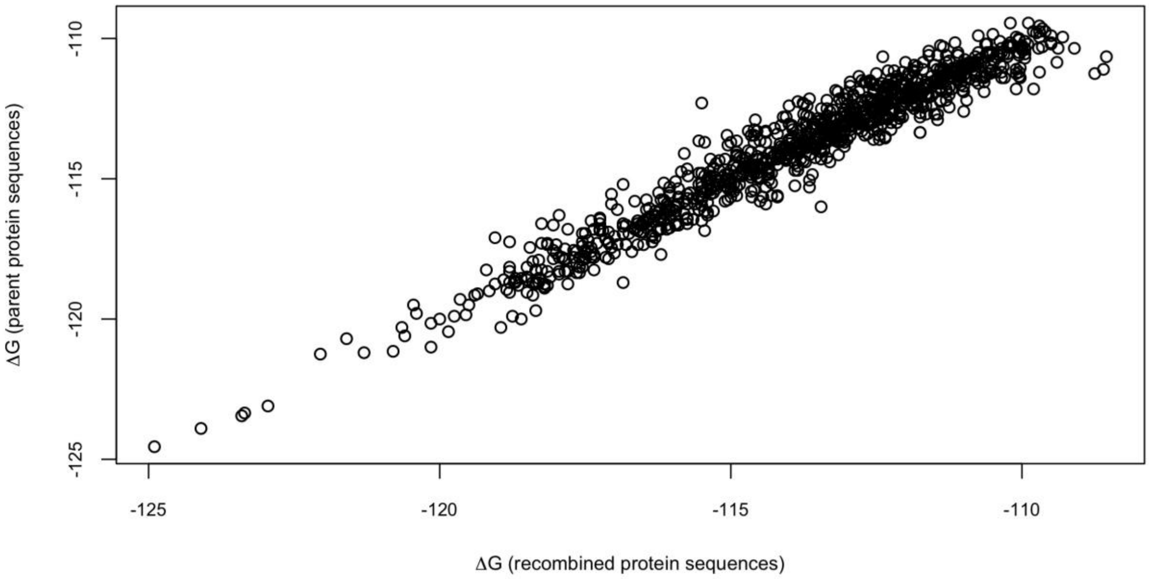

Figure S10. Folding free energy of recombined protein sequences as a function of folding free energy of parental protein sequences for the protein family TRXB. The plots show the mean of folding free energy of parent protein sequences (*y axis*) as a function of the mean of folding free energy of recombined protein sequences (*x axis*) in a total of 1,000 recombination events. Above plot considers recombination events at every breakpoint position (mean); correlation coefficient = 0.979, p value < $2.2e^{-16}$. Below plot, relationship considering recombination events with breakpoint position located in the middle of the sequences (correlation coefficient = 0.965, p value < $2.2e^{-16}$).

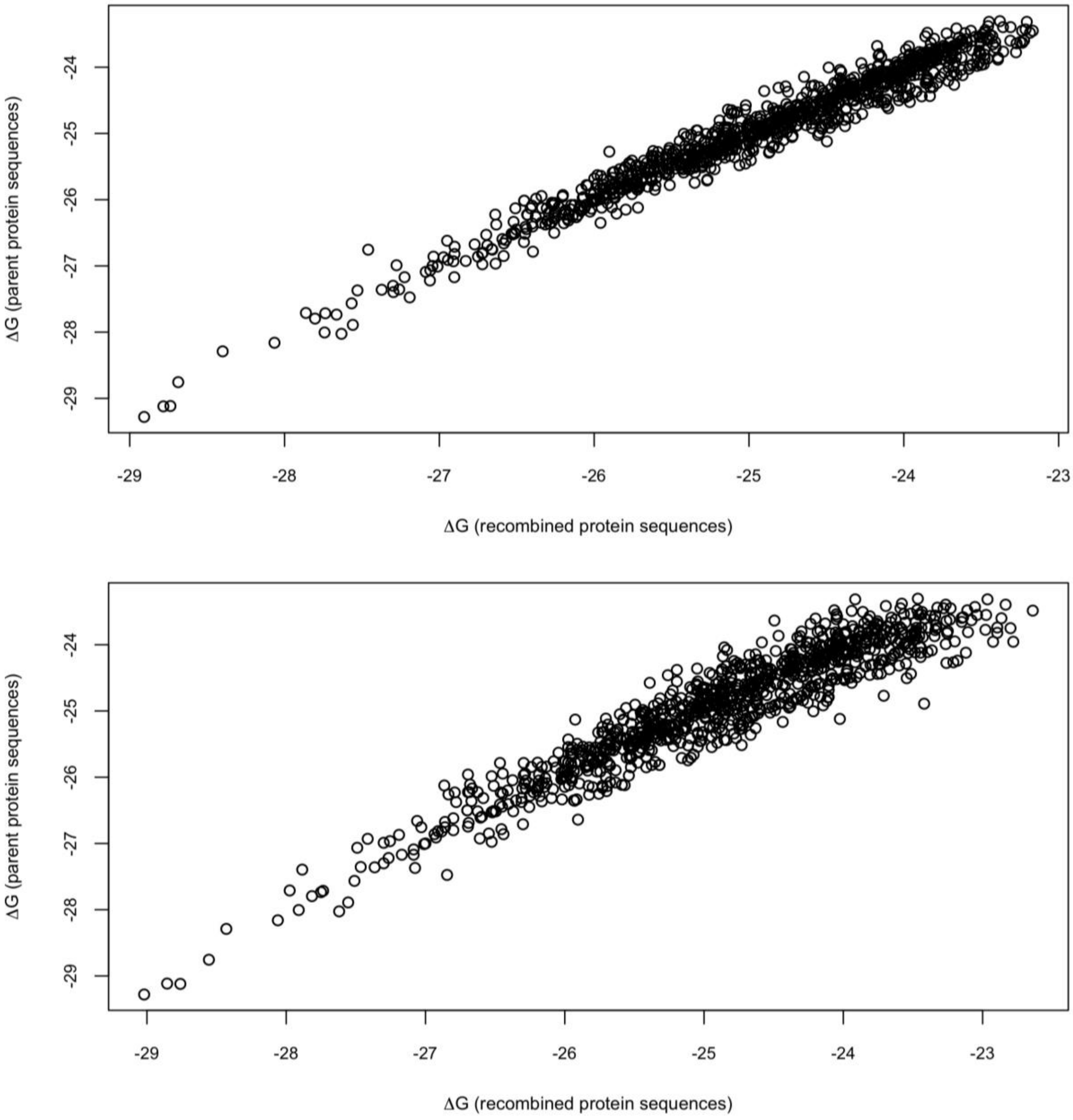

Figure S11. Variation of folding free energy between descendant proteins as a function of the variation of folding free energy between parental proteins for the protein family DDL. Every point refers to a recombination event. Left: Boxplots for intervals of folding free energy variation between the parental proteins. For every interval, the number of recombination events *N* falling in the interval and its fraction respect to the total (all intervals) number of recombination events (shown in parenthesis) is included. Results for recombination breakpoints in all the positions and in only the middle position are shown above and below, respectively.

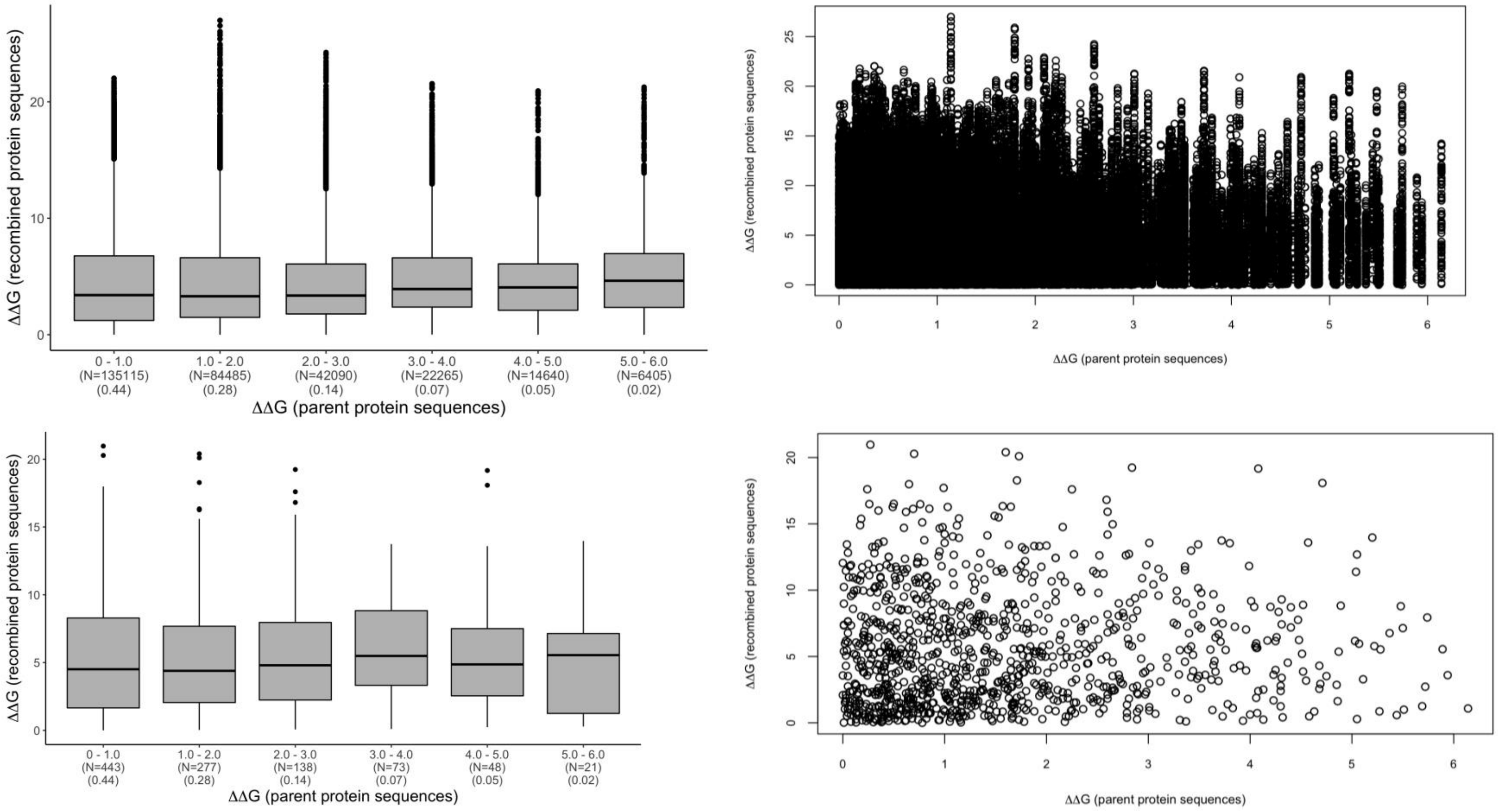

Figure S12. Variation of folding free energy between descendant proteins as a function of the variation of folding free energy between parental proteins for the protein family DNAK. Every point refers to a recombination event. Left: Boxplots for intervals of folding free energy variation between the parental proteins. For every interval, the number of recombination events *N* falling in the interval and its fraction respect to the total (all intervals) number of recombination events (shown in parenthesis) is included. Results for recombination breakpoints in all the positions and in only the middle position are shown above and below, respectively.

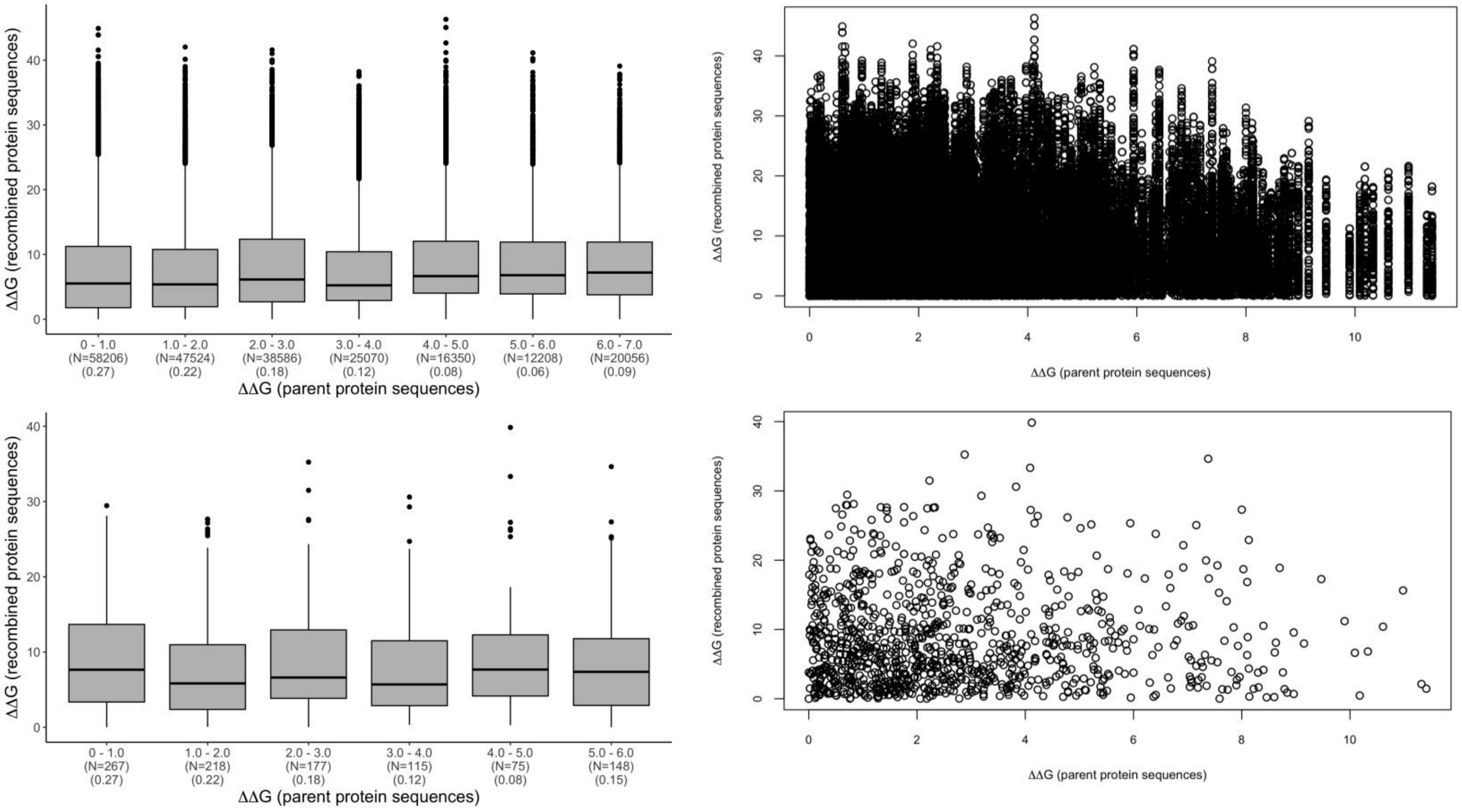

Figure S13. Variation of folding free energy between descendant proteins as a function of the variation of folding free energy between parental proteins for the protein family TPIS. Every point refers to a recombination event. Left: Boxplots for intervals of folding free energy variation between the parental proteins. For every interval, the number of recombination events *N* falling in the interval and its fraction respect to the total (all intervals) number of recombination events (shown in parenthesis) is included. Results for recombination breakpoints in all the positions and in only the middle position are shown above and below, respectively.

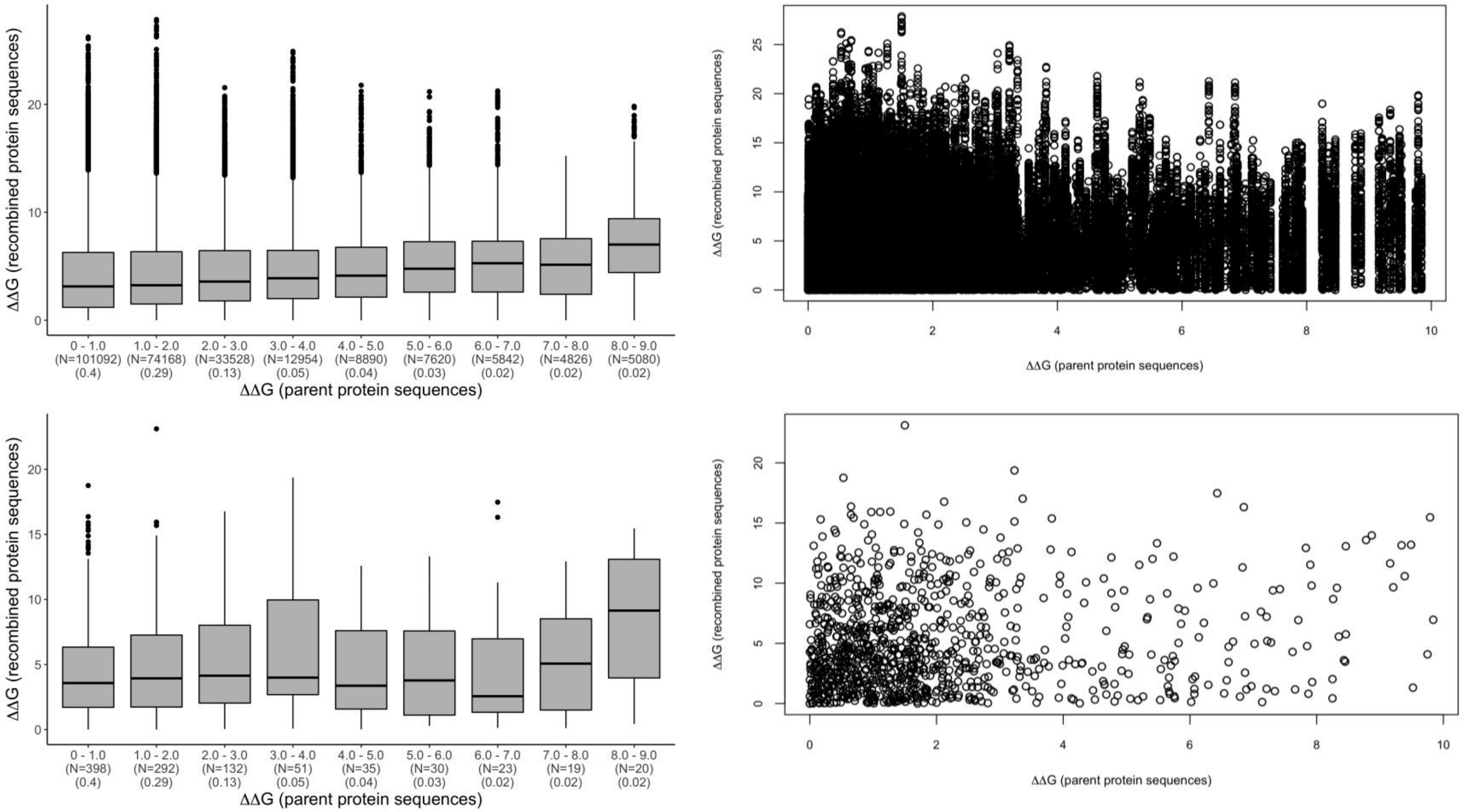

Figure S14. Variation of folding free energy between descendant proteins as a function of the variation of folding free energy between parental proteins for the protein family TRPA. Every point refers to a recombination event. Left: Boxplots for intervals of folding free energy variation between the parental proteins. For every interval, the number of recombination events *N* falling in the interval and its fraction respect to the total (all intervals) number of recombination events (shown in parenthesis) is included. Results for recombination breakpoints in all the positions and in only the middle position are shown above and below, respectively.

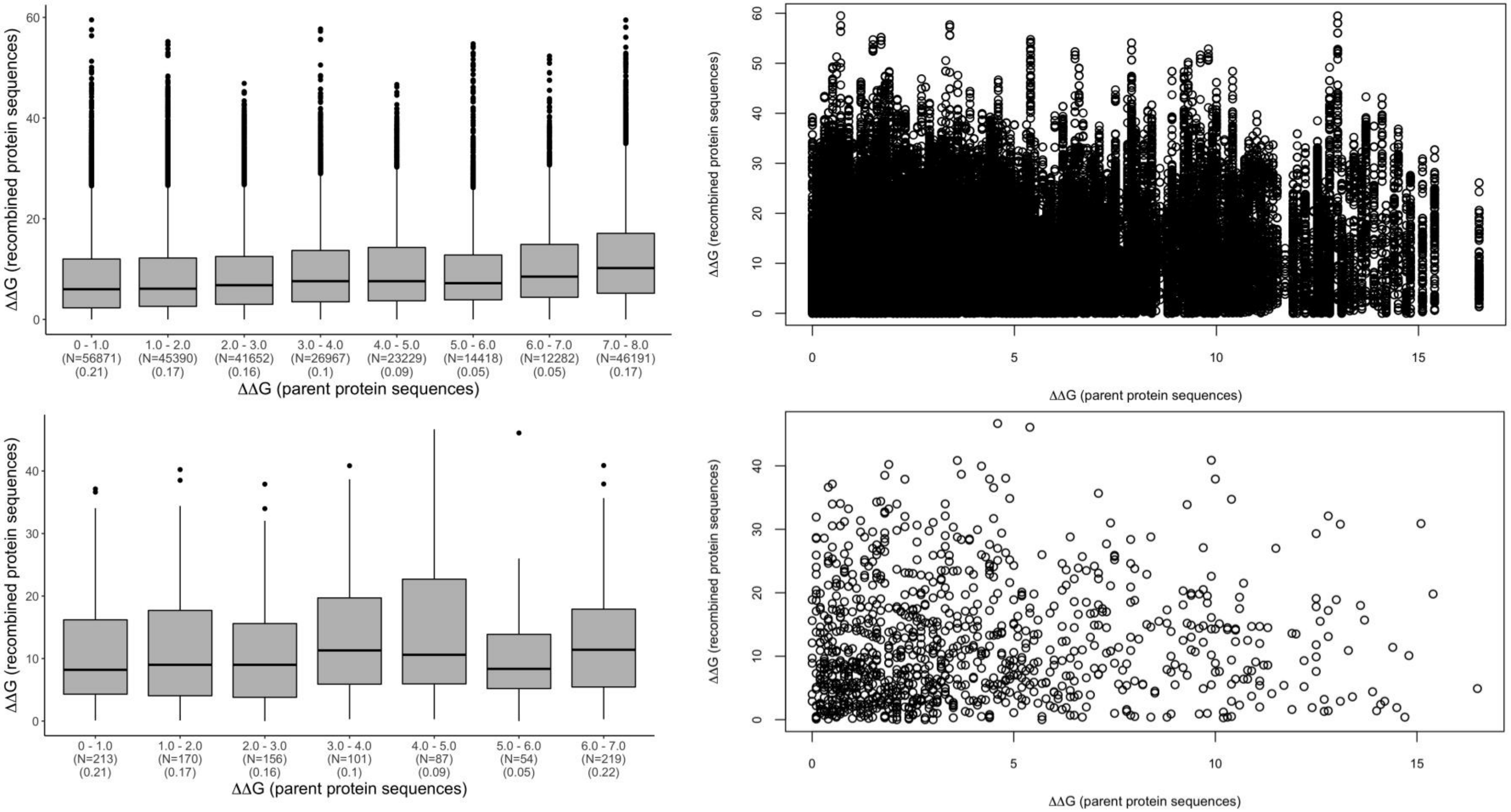

Figure S15. Variation of folding free energy between descendant proteins as a function of the variation of folding free energy between parental proteins for the protein family TRXB. Every point refers to a recombination event. Left: Boxplots for intervals of folding free energy variation between the parental proteins. For every interval, the number of recombination events *N* falling in the interval and its fraction respect to the total (all intervals) number of recombination events (shown in parenthesis) is included. Results for recombination breakpoints in all the positions and in only the middle position are shown above and below, respectively.

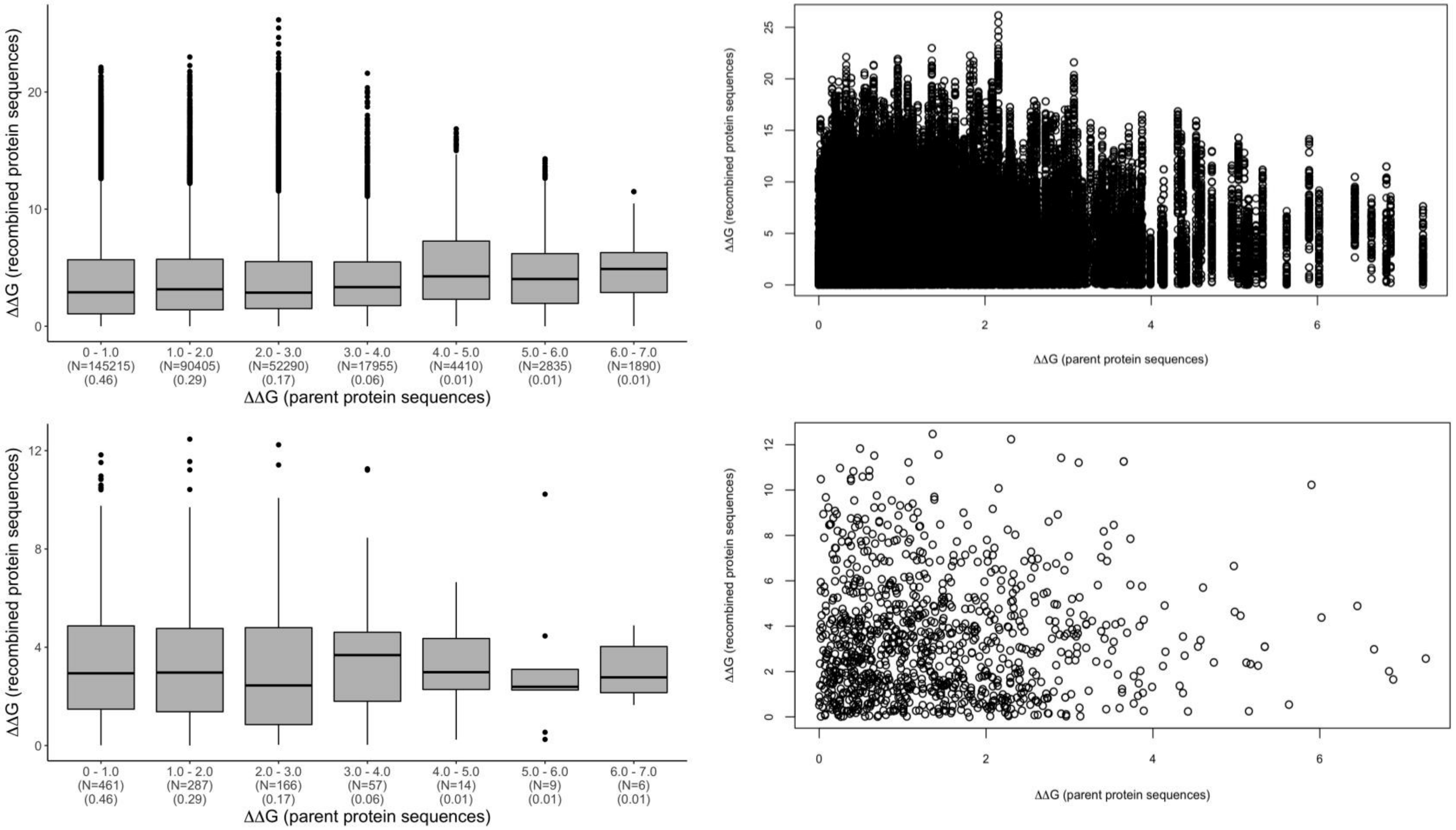

Figure S16. Acceptance rates of mutated and recombined sequences (breakpoints only located in the middle of sequences) in several protein families. The acceptation of a mutation or recombination event was defined as meeting $\Delta Gs \leq t\Delta Gr$, where ΔGs is the folding stability of the tested protein (i.e., generated by a mutation or recombination event), ΔGr is the folding stability of the real protein (Table 1) and t is a user-specified threshold. In this figure, the threshold is 0.95. The figure shows the acceptance rates of mutated sequences and recombined sequences, as well as the rates of recombination events accepting only one recombined sequence and both recombined sequences. Error bars correspond to the standard error of the mean of the respective mutation or recombination events. Results for the same analysis but focused on recombination events with breakpoints occurring in all the positions are shown in Figure 2.

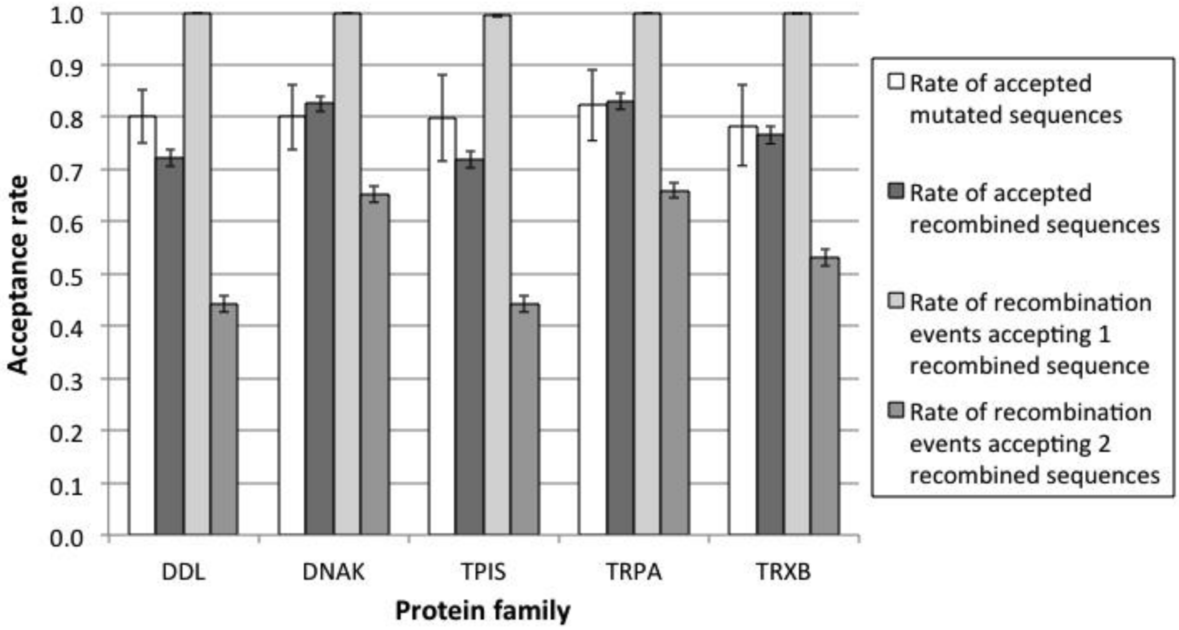

Figure S17. Evaluation of the protein folding stability caused by recombination and mutation events in several protein families. For every studied protein family, the figure shows the rate of accepted mutation events that increases the predicted protein stability, the rate of accepted recombination events producing both descendant (recombined) proteins more stable or unstable than both parental proteins and, the rate of recombination events producing one descendant protein more stable or unstable than both parental proteins. Results obtained considering a threshold of 0.95 to accept mutation and recombination events. This evaluation considered recombination events with breakpoints located in all the protein sites. Error bars indicate standard error of the mean of the corresponding mutation and recombination events. Results for the same analysis but focused on recombination events with breakpoints occurring only in the middle position of sequences are shown in Figure S18.

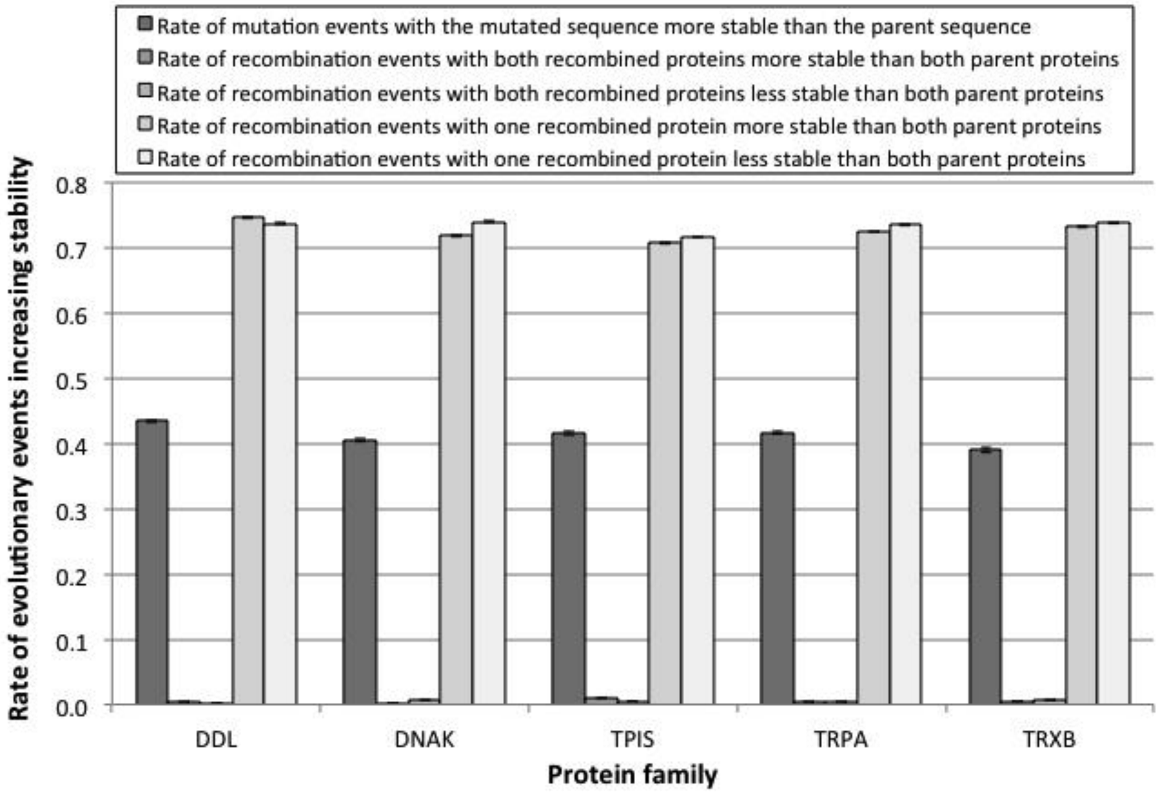

Figure S18. Evaluation of the protein folding stability caused by recombination (breakpoints only located in the middle of sequences) and mutation events in several protein families. For every studied protein family, the figure shows the rate of accepted mutation events that increases the predicted protein stability, the rate of accepted recombination events producing both descendant (recombined) proteins more stable or unstable than both parental proteins and, the rate of recombination events producing one descendant protein more stable or unstable than both parental proteins. Results obtained considering a threshold of 0.95 to accept mutation and recombination events. This evaluation considered recombination events with breakpoints located in all the protein sites. Error bars indicate standard error of the mean of the corresponding mutation and recombination events. Results for the same analysis but focused on recombination events with breakpoints occurring in all the positions are shown in Figure S17.

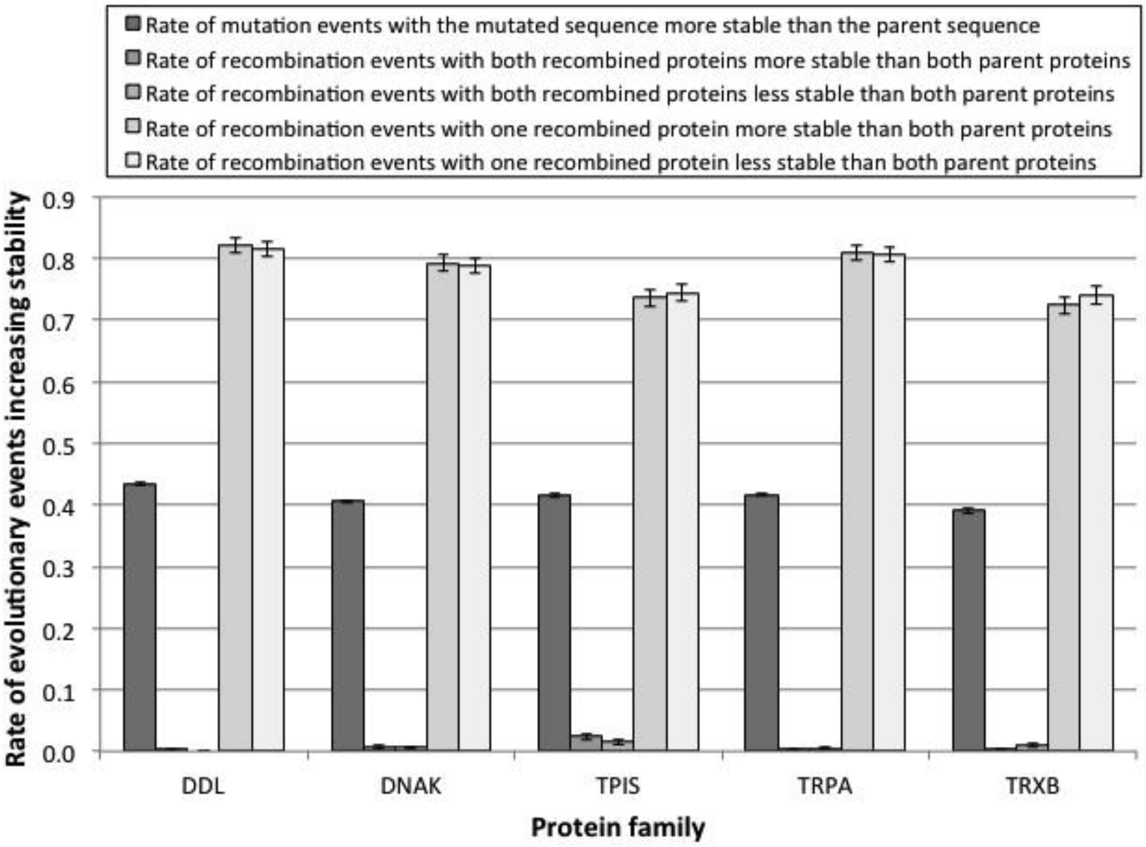

Figure S19. Rates of accepted mutated and recombined sequences that are more stable or unstable than their parent sequences for different protein families. The figure shows the rate of mutated sequences more stable than their parent sequences and the rates of recombined (descendant) sequences that are more stable or unstable than both or one of the parental sequences. Results obtained considering a threshold of 0.95 to accept mutation and recombination events. Error bars indicate standard error of the mean of the corresponding mutation and recombination events. Above: Recombination breakpoints located in all the positions. Below: Recombination breakpoints located only in the middle of sequences.

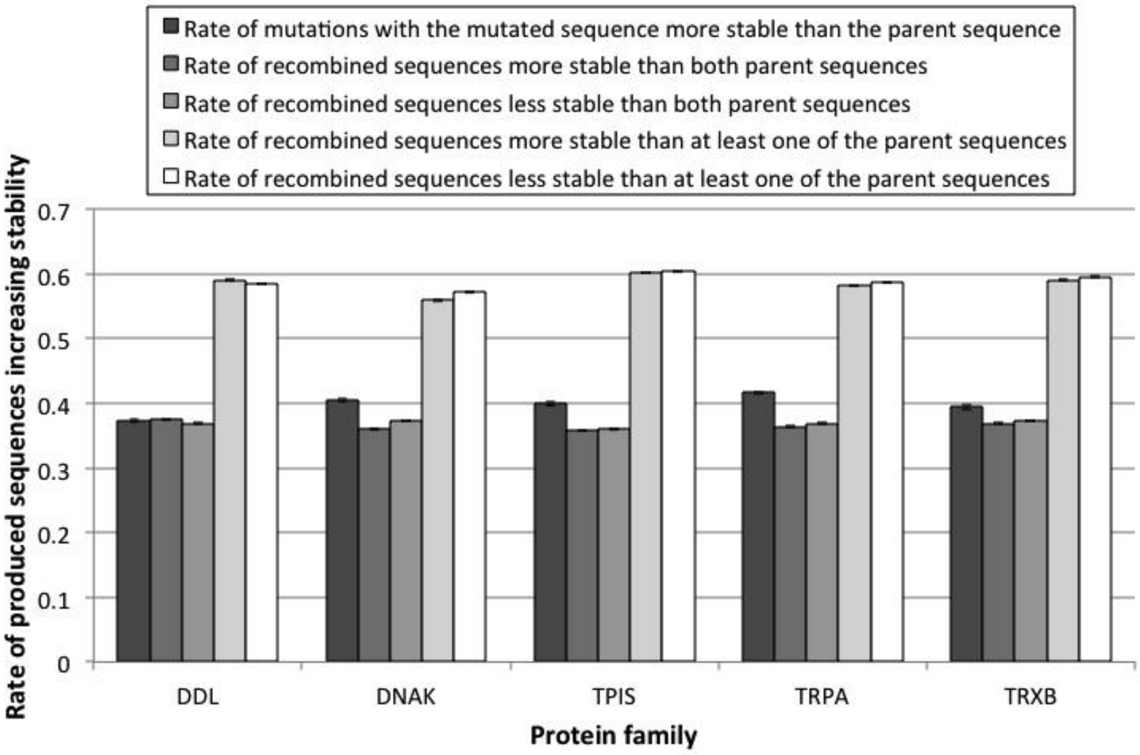

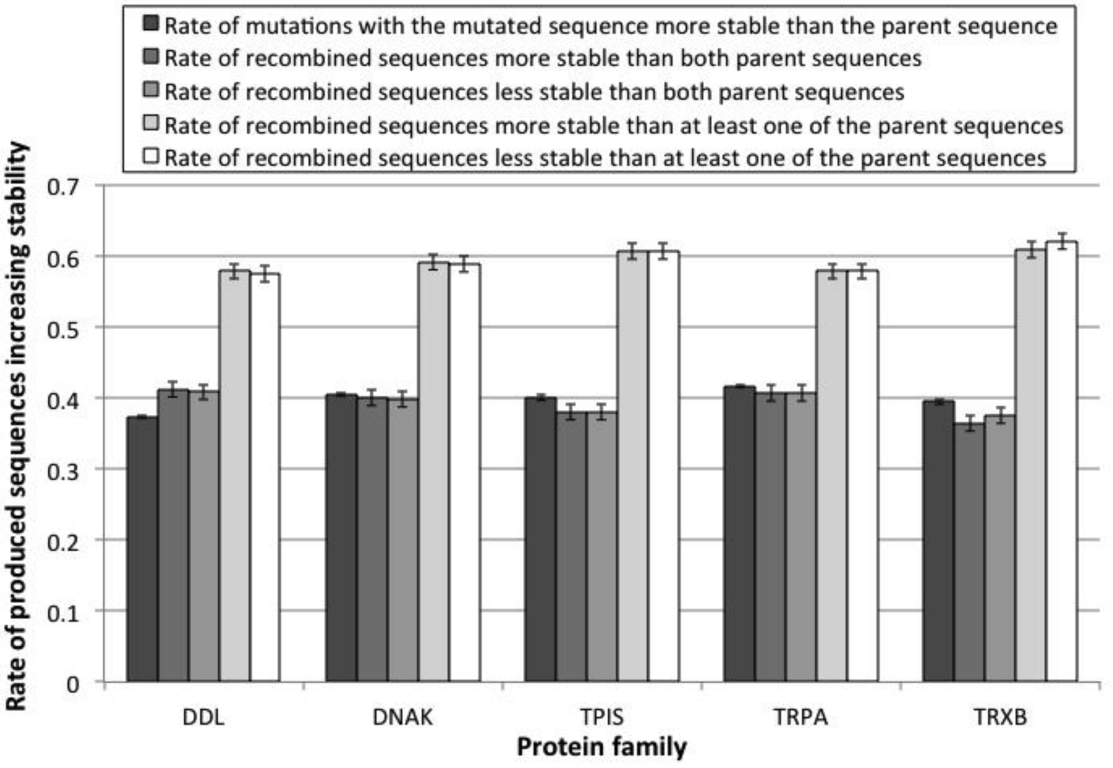

Figure S20. Rates of accepted mutated and recombined sequences (breakpoints only located in the middle of sequences) that are more stable or unstable than their parent sequences at diverse selection levels. The figure shows the rate of mutated sequences more stable than their parent sequences and the rates of recombined (descendant) sequences that are more stable or unstable than both or one of the parental sequences. Results based on simulations of the DDL protein family. Error bars indicate standard error of the mean of the corresponding mutation and recombination events. This evaluation considers recombination events with breakpoints only located in the middle of sequences. Results for the same analysis but focused on recombination events with breakpoints occurring in all the positions are shown in Figure 4.

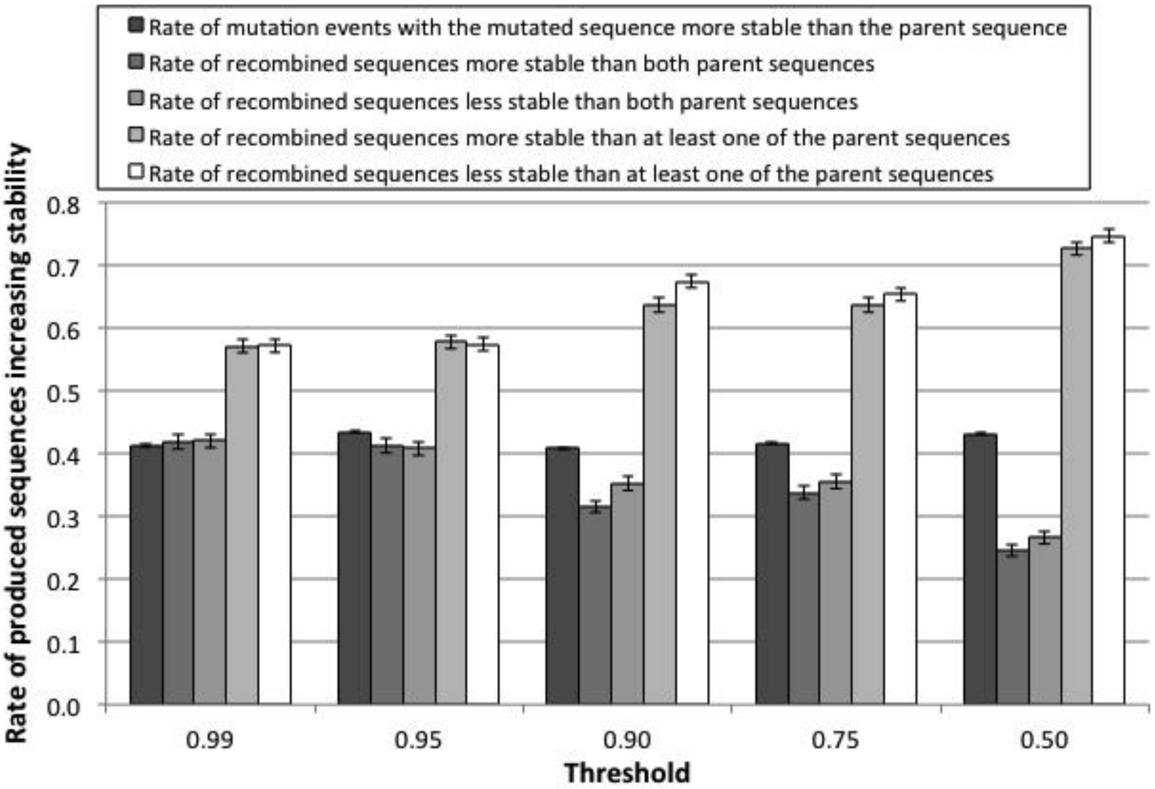

Figure S21. Influence of sequence identity between parental sequences on the folding free energy caused by recombination in the protein family DNAK. The figure shows the folding free energy variation produced by recombination (ΔΔG) between recombinant (parental) and recombined (descendant) sequences. Negative values mean that the two sequences before recombining are more stable (mean) than the two sequences after recombining (mean), and the opposite for positive values, as a function of the sequence identity (shown on the right by intervals) between the parental sequences. Results based on a selection threshold of 0.95. The above plots refer to recombination events occurring in all the breakpoint positions (mean) and plots below refer to recombination events with breakpoint position only located in the middle of the sequences.

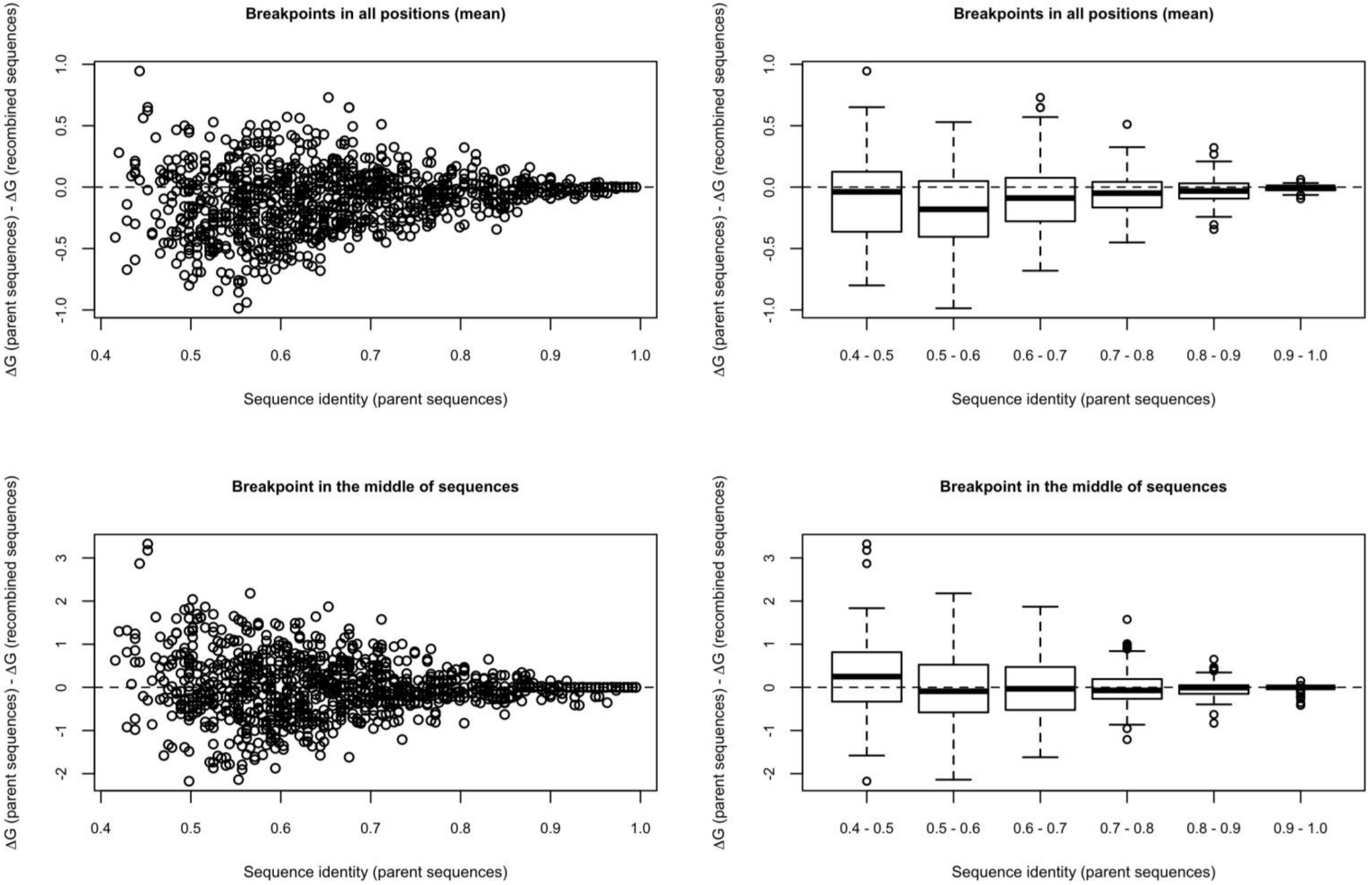

Figure S22. Influence of sequence identity between parental sequences on the folding free energy caused by recombination in the protein family TPIS. The figure shows the folding free energy variation produced by recombination (ΔΔG) between recombinant (parental) and recombined (descendant) sequences. Negative values mean that the two sequences before recombining are more stable (mean) than the two sequences after recombining (mean), and the opposite for positive values, as a function of the sequence identity (shown on the right by intervals) between the parental sequences. Results based on a selection threshold of 0.95. The above plots refer to recombination events occurring in all the breakpoint positions (mean) and plots below refer to recombination events with breakpoint position only located in the middle of the sequences.

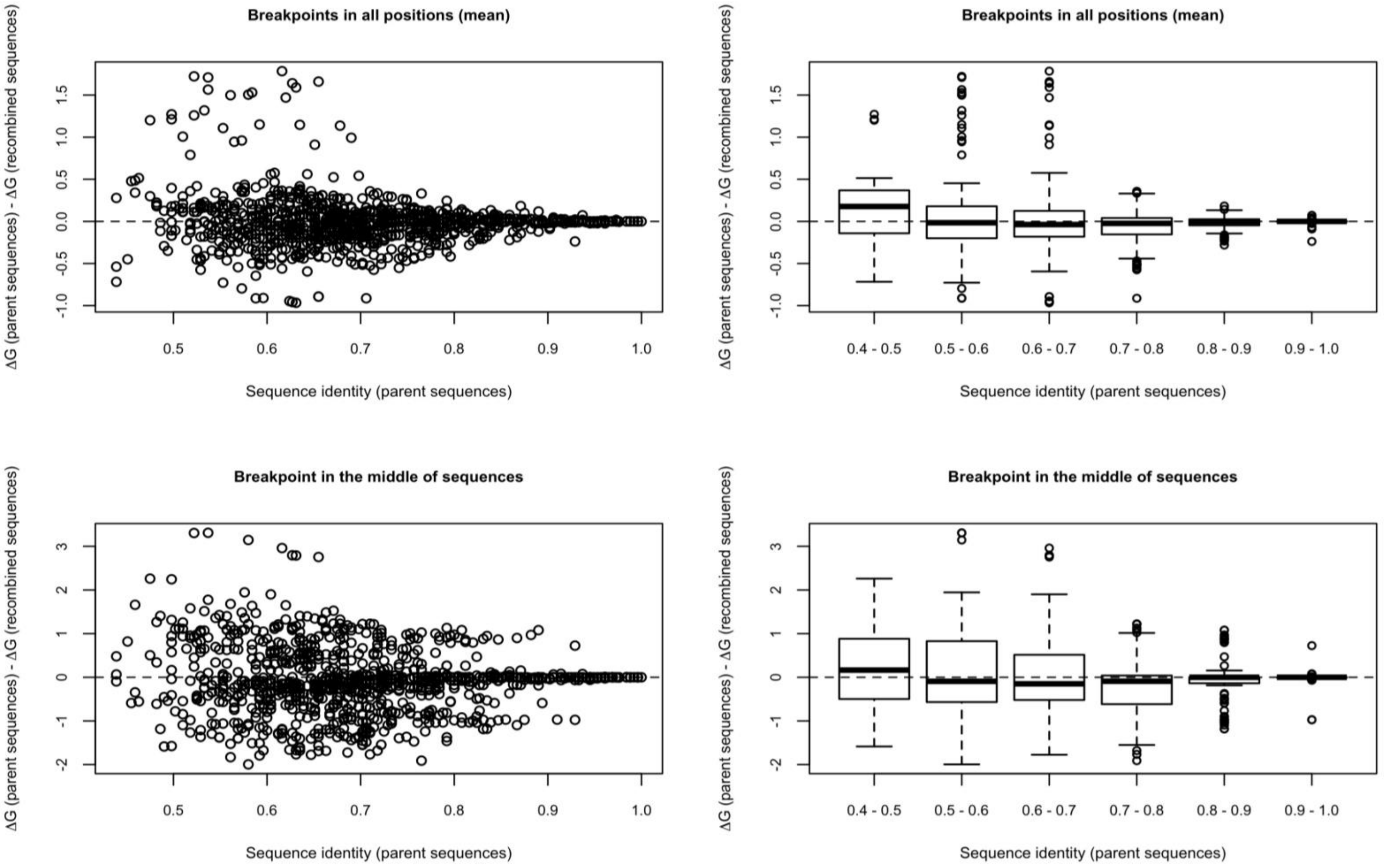

Figure S23. Influence of sequence identity between parental sequences on the folding free energy caused by recombination in the protein family TRPA. The figure shows the folding free energy variation produced by recombination (ΔΔG) between recombinant (parental) and recombined (descendant) sequences. Negative values mean that the two sequences before recombining are more stable (mean) than the two sequences after recombining (mean), and the opposite for positive values, as a function of the sequence identity (shown on the right by intervals) between the parental sequences. Results based on a selection threshold of 0.95. The above plots refer to recombination events occurring in all the breakpoint positions (mean) and plots below refer to recombination events with breakpoint position only located in the middle of the sequences.

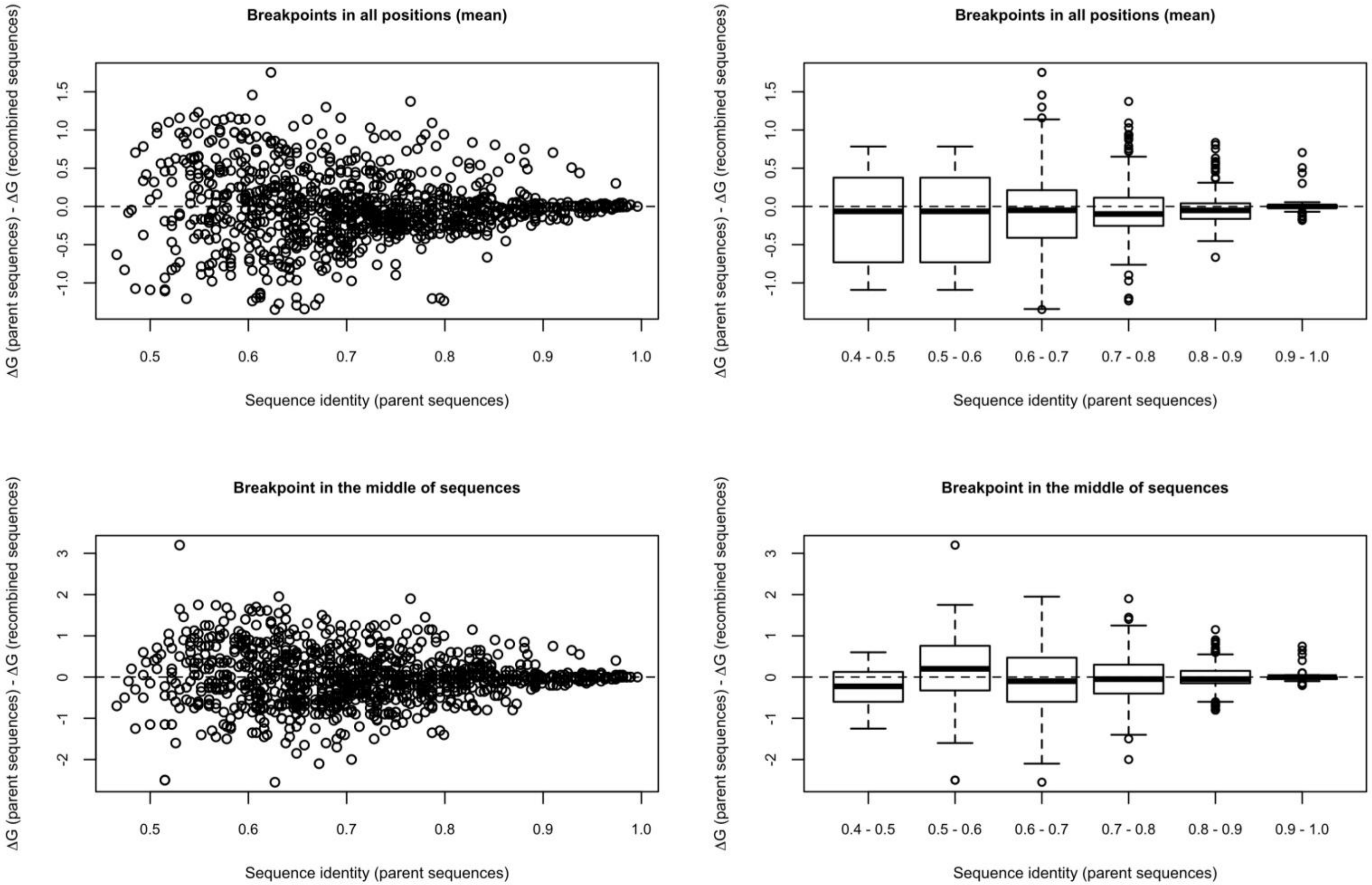

Figure S24. Influence of sequence identity between parental sequences on the folding free energy caused by recombination in the protein family TRXB. The figure shows the folding free energy variation produced by recombination (ΔΔG) between recombinant (parental) and recombined (descendant) sequences. Negative values mean that the two sequences before recombining are more stable (mean) than the two sequences after recombining (mean), and the opposite for positive values, as a function of the sequence identity (shown on the right by intervals) between the parental sequences. Results based on a selection threshold of 0.95. The above plots refer to recombination events occurring in all the breakpoint positions (mean) and plots below refer to recombination events with breakpoint position only located in the middle of the sequences.

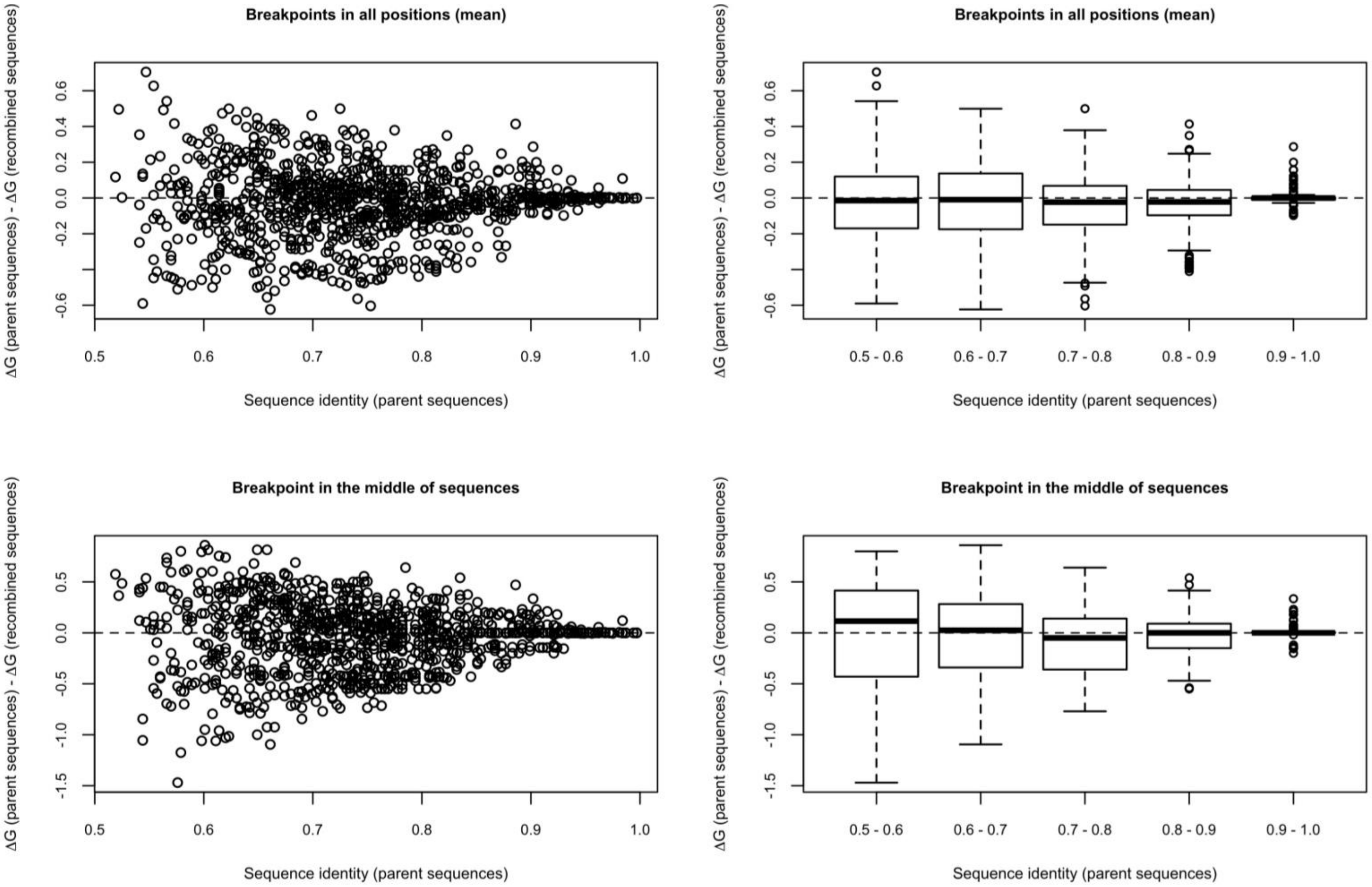

Figure S25. Folding free energy of parent and descendant protein sequences involved in real recombination events. Folding free energy (ΔG) of protein sequences involved in real recombination events detected in 4 illustrative datasets from viruses (Table S1). Each column refers to a recombination event (note that the Dataset 1 presented 3 recombination events) with the detected breakpoints shown in the x-axis. The folding stability of the PDB representative for the studied proteins of every dataset is shown with a dashed line.

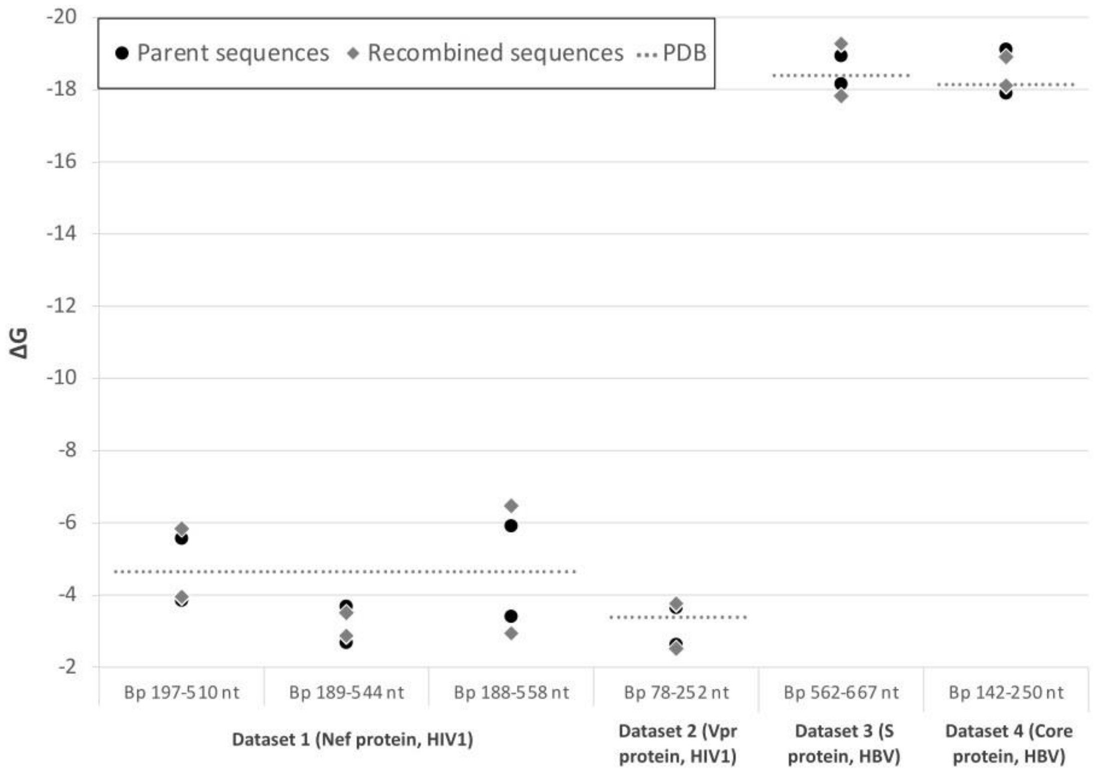

Figure S26. Influence of sequence identity between parental sequences on the folding free energy caused by illustrative real recombination events. The figure shows the folding free energy variation produced by recombination (ΔΔG) between recombinant (parental) and recombined (descendant) sequences in real recombination events detected in 4 illustrative datasets from viruses (Table S1).

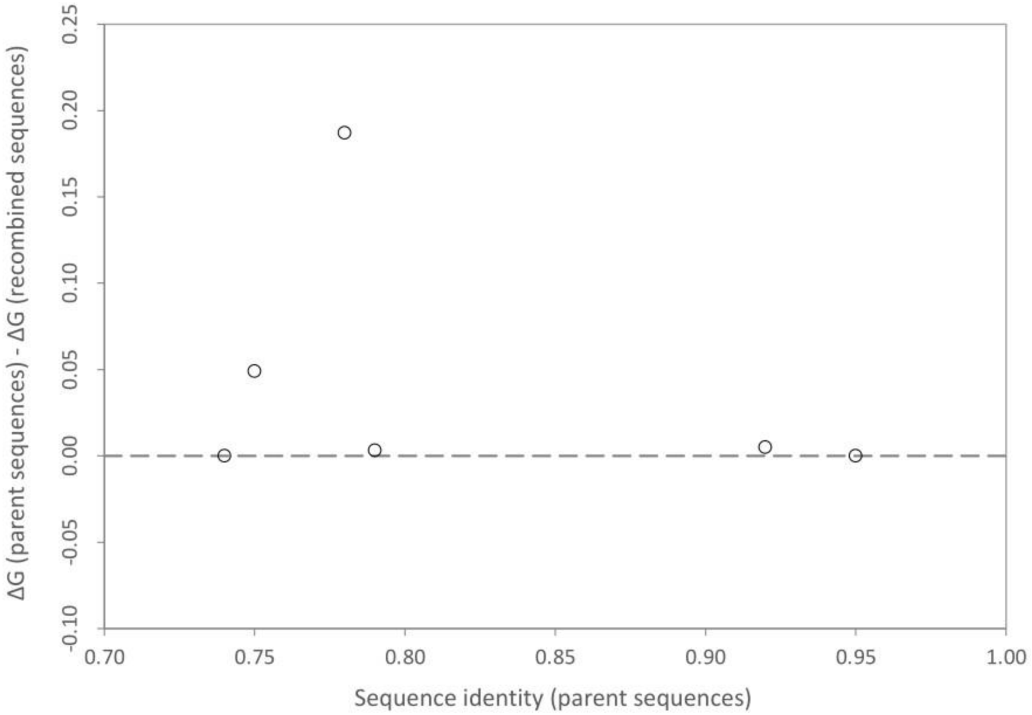

Figure S27. Folding free energy of protein sequences simulated upon a phylogenetic tree under empirical and structurally constrained substitution models for diverse protein families. Folding free energy (ΔG) of protein sequences simulated without recombination under the best-fitting empirical substitution model (Table 1) (squares) and the structurally constrained substitution (SCS) model (circles) at different times (internal and tip nodes of the phylogenetic tree). The root (time to root = 0) corresponds to the extant PDB protein structure chosen as a representative structure of the protein family. Error bars correspond to the 95% confidence interval (CI) of the mean from 100 computer simulations. Comparing with the ΔG of the extant PDB protein structure, the empirical substitution model generates unrealistically unstable protein sequences. Results for the protein family DDL are shown above while results for the other protein families (TPIS, DNAK, TRPA and TRXB) are shown below.

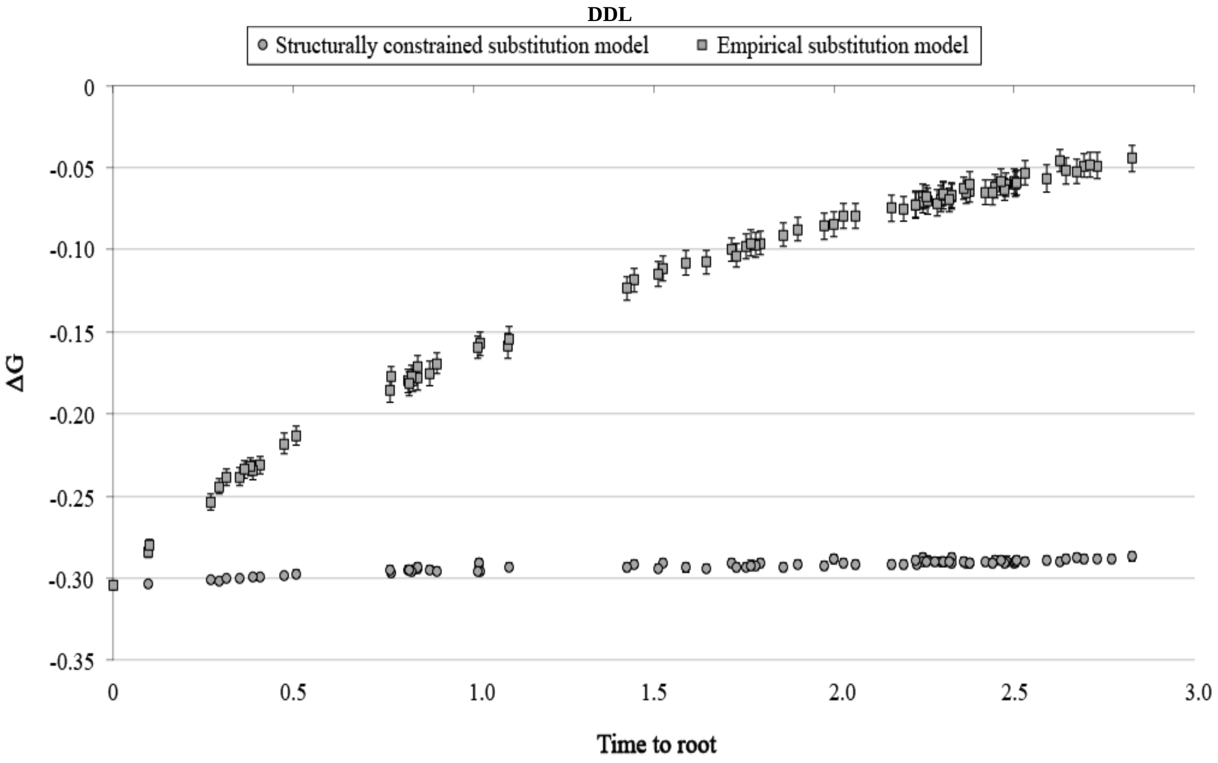

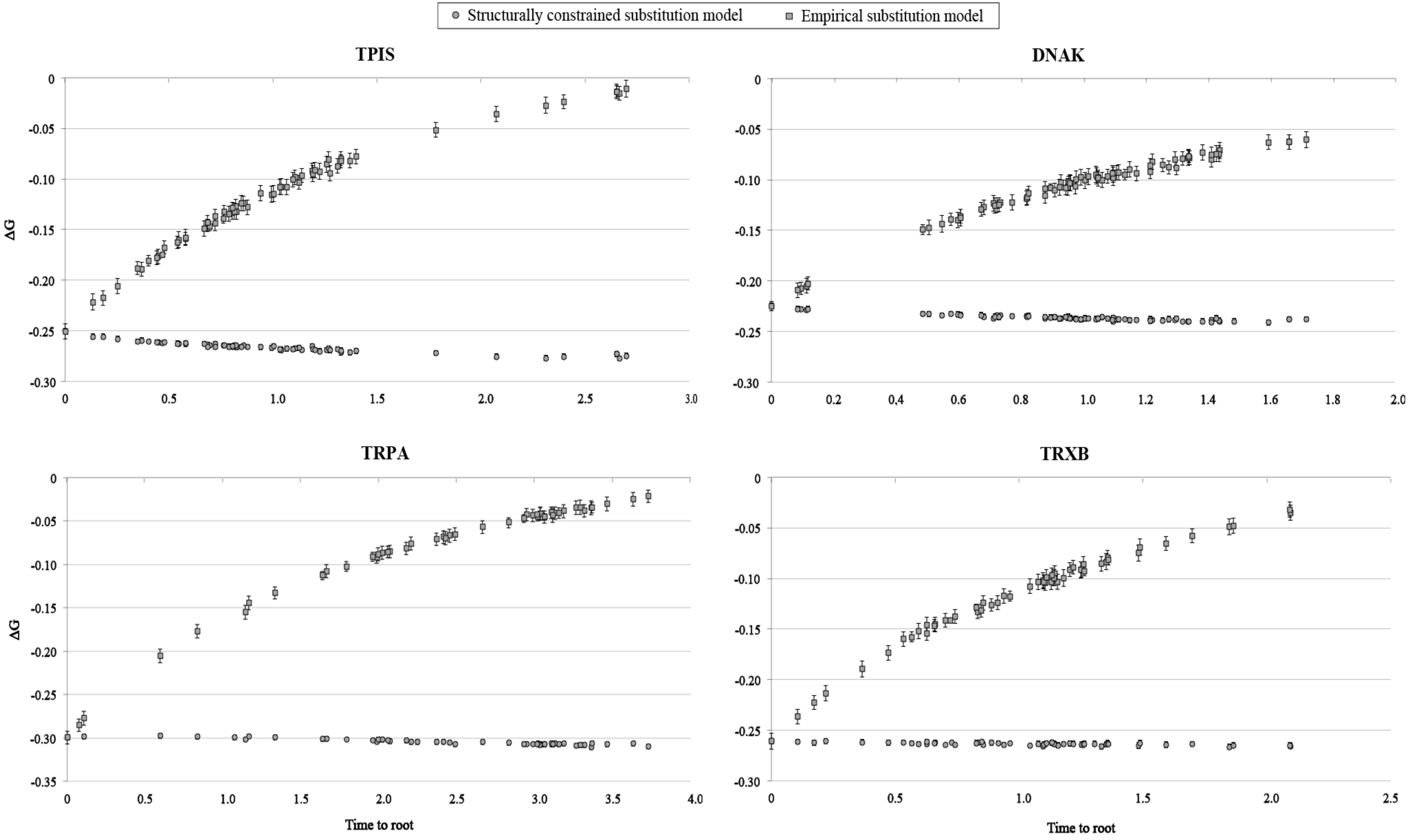

Structurally constrained substitution model
Empirical substitution model
TPIS
DNAK
TRPA
TRXB
ΔG
Time to root

Figure S28. Folding free energy of TPIS, DNAK, TRPA and TRXB proteins simulated upon coalescent trees with diverse combinations of population substitution and recombination rates. Folding free energy (ΔG) of proteins simulated upon coalescent trees previously simulated under a variety of combinations of population substitution rate (θ) and population recombination rate (ρ) and where the protein sequences evolved under the best-fitting empirical substitution model (Table 1). The dashed line corresponds to the ΔG of the extant protein structure of the protein family (Table 1). Error bars represent the 95% confidence interval among the mean of 100 computer simulations. Results for the protein family DDL are presented in Figure 6.

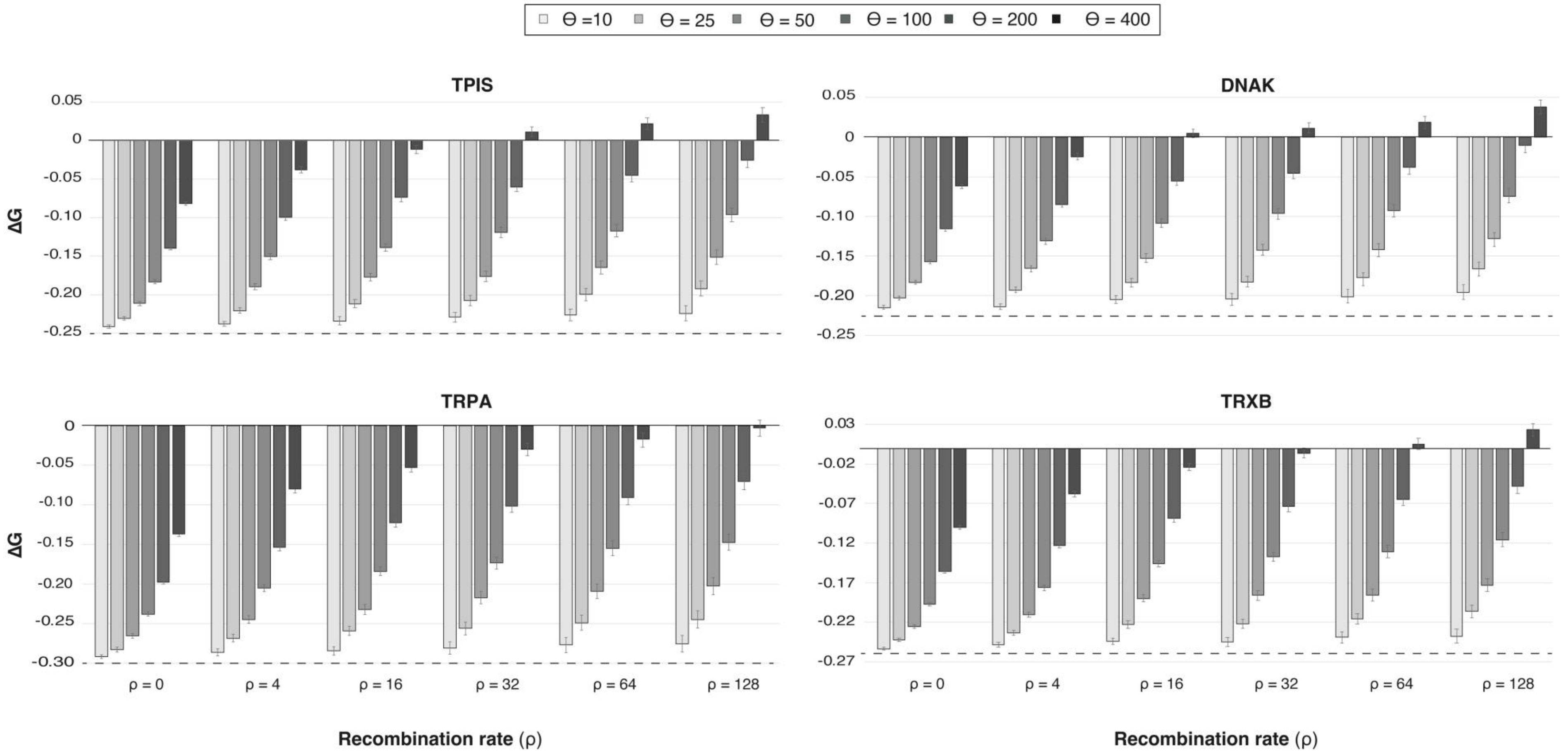

Figure S29. Variation of protein folding stability between parental (recombinant) and descendant (recombined) proteins as a function of the sequence identity between the parental proteins for the DDL protein family. Every point represents a recombination event that was simulated under a particular combination of substitution and recombination rates.

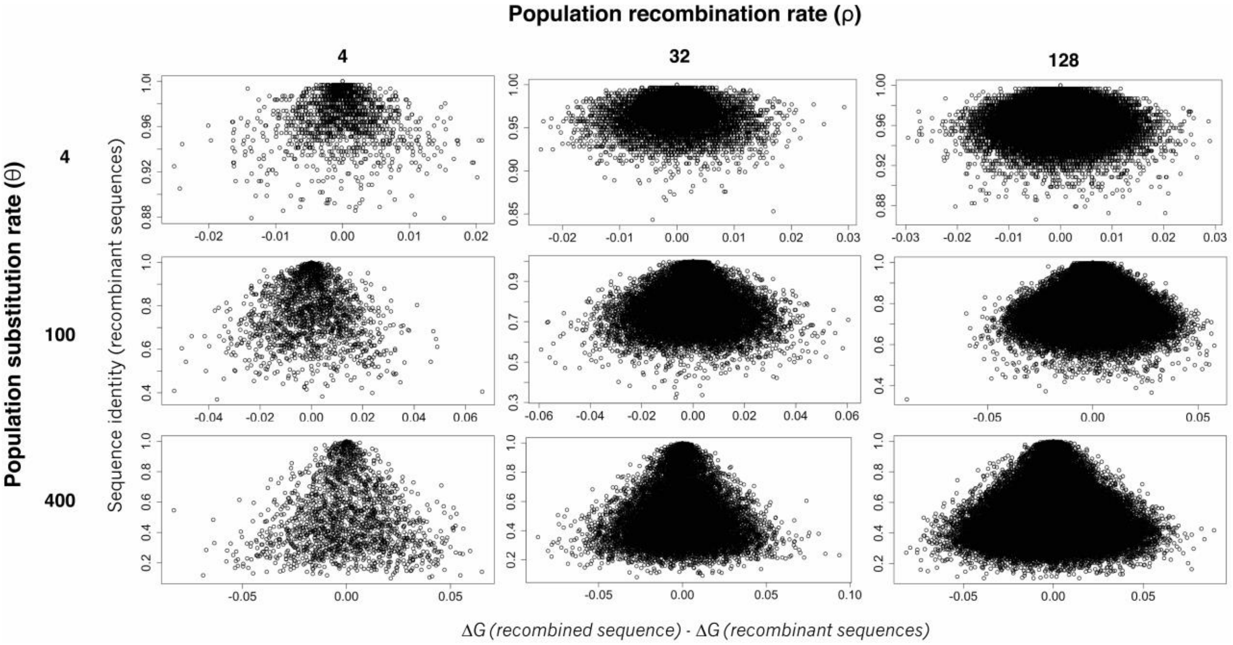

Figure S30. Variation of protein folding stability between parental (recombinant) and descendant (recombined) proteins as a function of the sequence identity between the parental proteins for the DNAK protein family. Every point represents a recombination event that was simulated under a particular combination of substitution and recombination rates.

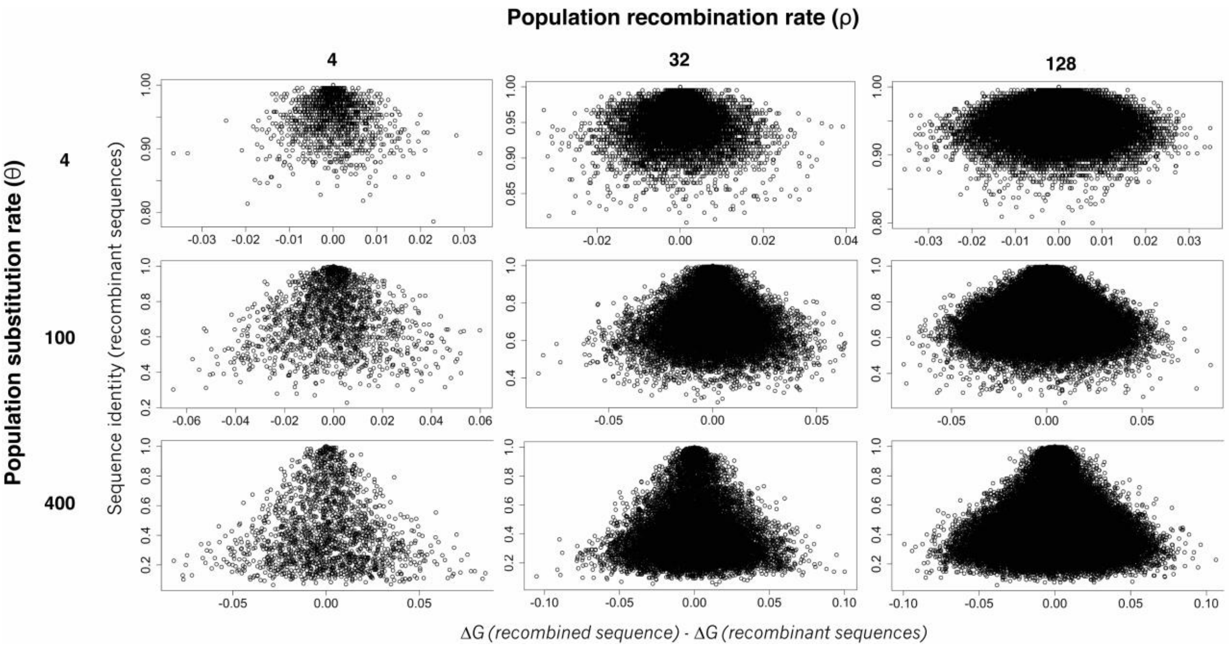

Figure S31. Variation of protein folding stability between parental (recombinant) and descendant (recombined) proteins as a function of the sequence identity between the parental proteins for the TPIS protein family. Every point represents a recombination event that was simulated under a particular combination of substitution and recombination rates.

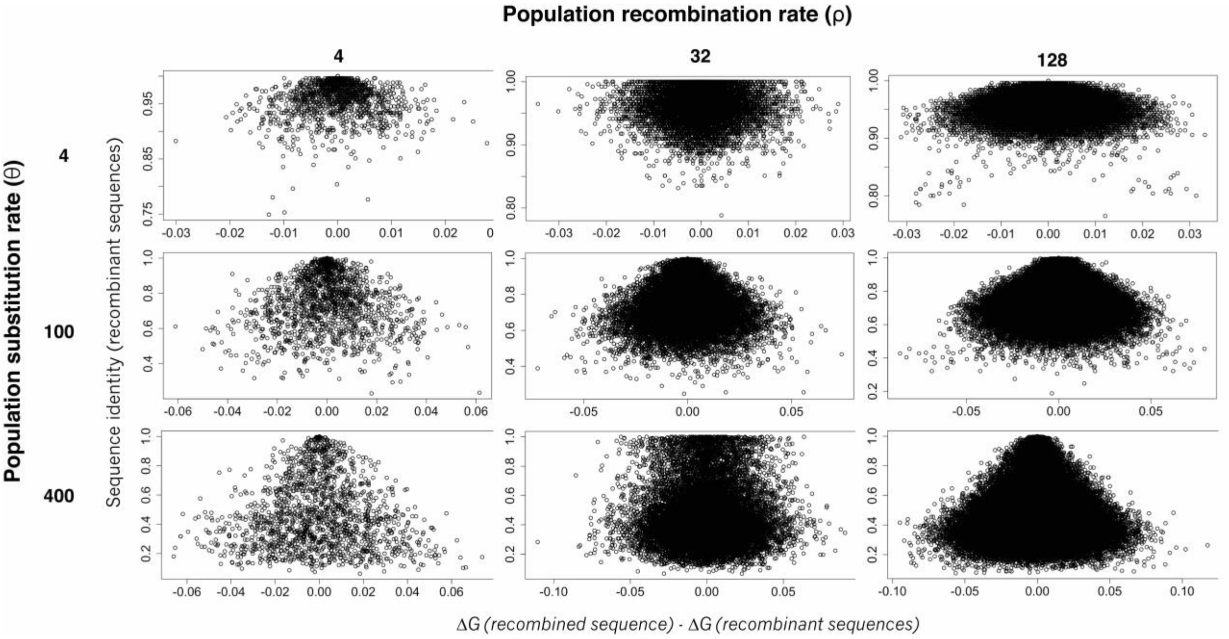

Figure S32. Variation of protein folding stability between parental (recombinant) and descendant (recombined) proteins as a function of the sequence identity between the parental proteins for the TRPA protein family. Every point represents a recombination event that was simulated under a particular combination of substitution and recombination rates.

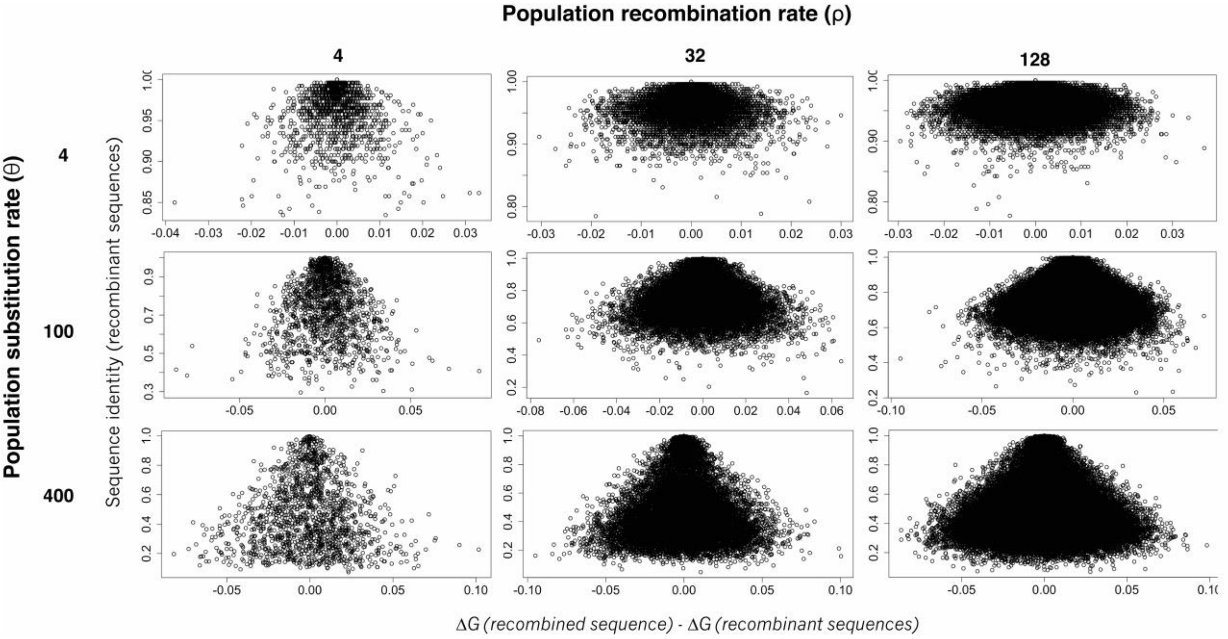

Figure S33. Variation of protein folding stability between parental (recombinant) and descendant (recombined) proteins as a function of the sequence identity between the parental proteins for the TRXB protein family. Every point represents a recombination event that was simulated under a particular combination of substitution and recombination rates.

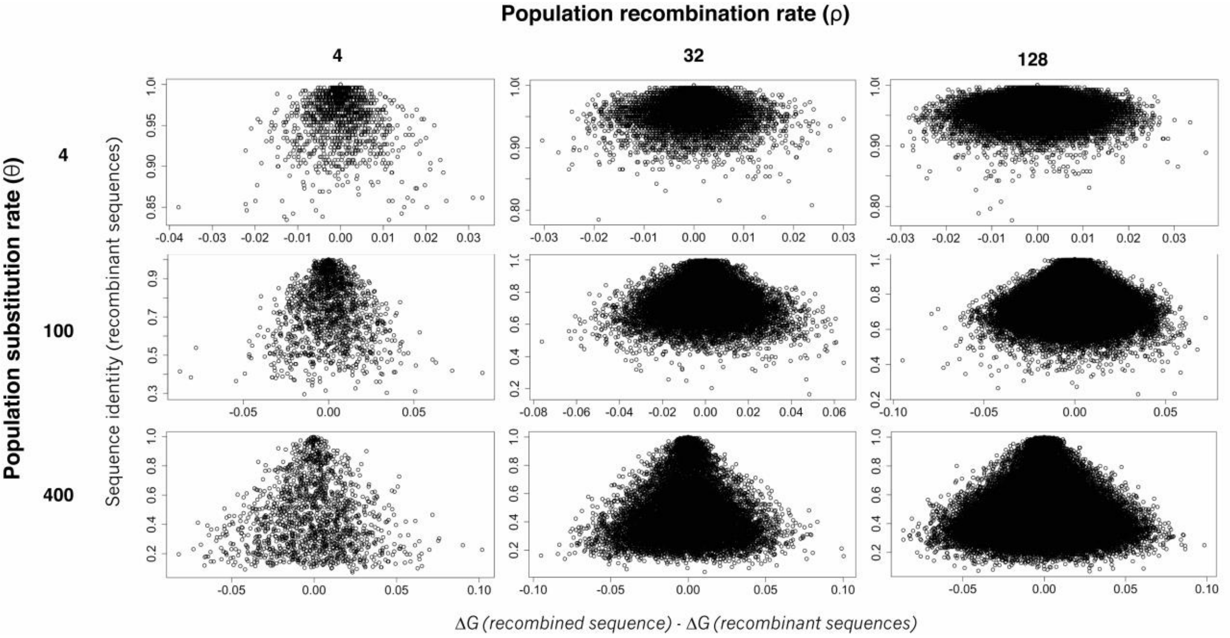

Table S1. Illustrative examples of real data and results from their recombination analyses. For each dataset, the table shows protein and organism, *Popset* code, representative protein (PDB structure), sequence length (number of amino acids), sample size (number of sequences), sequence identity at amino acid and nucleotide levels, recombination tests indicating the presence of a recombination event (including *P value*), Genbank code of sequences that recombined (parents), sequence identity at amino acid and nucleotide levels between the parent sequences, recombination breakpoints and reference of the study dataset. Note that the first dataset presented 3 recombination events.

**Protein and Organism**	**Popset code**	**PDB code and chain**	**Protein length**	**Sample size**	**Seq Id (aa)**	**Seq Id (nt)**	**Tests indicating recombination (*P value*)**	**Parents (Genbank code)**	**Parents Id (aa)**	**Parents Id (nt)**	**Bp (nt)**	**Reference**
Nef, HIV1	1725349635	6URI, C	186	101	0.81	0.85	MaxChi (1.80x10^{-4})<br>Chimaera (1.83x10^{-2})<br>SiScan (2.50x10^{-3})<br>3Seq (6.90x10^{-3})	MK333880.1<br>MK333860.1	0.78	0.80	197, 510	(Acharya, et al., 2020)
							MaxChi (1.33x10^{-2})<br>Chimaera (6.16x10^{-4})<br>3Seq (8.17x10^{-4})	MK333831.1<br>MK333849.1	0.79	0.83	189, 544	
							MaxChi (9.83x10^{-4})<br>Chimaera (3.41x10^{-2})<br>3Seq (4.87x10^{-2})	MK333824.1<br>MK333890.1	0.75	0.79	188, 558	
Vpr, HIV1	170778936	1M8L, A	96	41	0.86	0.89	MaxChi (4.54x10^{-4})<br>3Seq (2.07x10^{-2})	EU518114.1<br>EU518095.1	0.74	0.82	78, 252	(Miura, et al., 2008)
S protein, HBV	511774227	7TUK, A	226	52	0.97	0.98	GENECONV (2.41x10^{-2})<br>3Seq (5.13x10^{-3})	KC836778.1<br>KC836821.1	0.92	0.94	562, 667	(Lin, et al., 2016)
Core protein, HBV	30525184	7ABL, C	183	47	0.97	0.95	MaxChi (2.11x10^{-2})<br>SiScan (3.10x10^{-3})<br>3Seq (3.88x10^{-2})	AY269073.1<br>AY269079.1	0.95	0.96	142, 250	(Jazayeri, et al., 2004)

Table S2. Frequency of recombination events producing a descendant protein with higher, similar or lower folding stability than that displayed by the parental proteins. For several combinations of population substitution rate (θ) and population recombination rate (ρ), and for each studied protein family, the table shows the percentage of recombination events where the descendant protein presents a higher, intermediate (between the stability of the parental proteins; which was the most frequently observed situation) or lower folding stability than that of the parental proteins.

DDL

	**θ = 10**	**θ = 100**	**θ = 400**
	**Stable / Intermediate / Instable**	**Stable / Intermediate / Instable**	**Stable / Intermediate / Instable**
**ρ = 4**	15 / 70 / 15	22 / 56 / 22	24 / 52 / 24
**ρ = 32**	16 / 68 / 16	23 / 54 / 23	25 / 50 / 25
**ρ = 128**	18 / 64 / 18	24 / 52 / 24	25 / 50 / 25

TPIS

	**θ = 10**	**θ = 100**	**θ = 400**
	**Stable / Intermediate / Instable**	**Stable / Intermediate / Instable**	**Stable / Intermediate / Instable**
**ρ = 4**	16 / 68 / 16	23 / 54 / 23	26 / 48 / 26
**ρ = 32**	17 / 66 / 17	23 / 54 / 23	25 / 50 / 25
**ρ = 128**	18 / 64 / 18	24 / 52 / 24	25 / 50 / 25

DNAK

	**θ = 10**	**θ = 100**	**θ = 400**
	**Stable / Intermediate / Instable**	**Stable / Intermediate / Instable**	**Stable / Intermediate / Instable**
**ρ = 4**	15 / 70 / 15	23 / 54 / 23	23 / 54 / 23
**ρ = 32**	16 / 68 / 16	23 / 54 / 23	24 / 25 / 51
**ρ = 128**	18 / 64 / 18	24 / 52 / 24	25 / 50 / 25

TRPA

	**θ = 10**	**θ = 100**	**θ = 400**
	**Stable / Intermediate / Instable**	**Stable / Intermediate / Instable**	**Stable / Intermediate / Instable**
**ρ = 4**	14 / 71 / 15	22 / 53 / 25	24 / 51 / 25
**ρ = 32**	16 / 68 / 16	24 / 53 / 23	25 / 50 / 25
**ρ = 128**	18 / 64 / 18	24 / 52 / 24	25 / 50 / 25

	TRXB		
	**θ = 10**	**θ = 100**	**θ = 400**
	**Stable / Intermediate / Instable**	**Stable / Intermediate / Instable**	**Stable / Intermediate / Instable**
**ρ = 4**	14 / 70 / 16	23 / 54 / 23	10 / 80 / 10
**ρ = 32**	15 / 71 / 14	22 / 55 / 23	10 / 80 / 10
**ρ = 128**	18 / 64 / 18	24 / 52 / 24	11 / 78 / 11

References

Acharya, A., *et al.* Variability in HIV-1 Integrase Gene and 3′-Polypurine Tract Sequences in Cameroon Clinical Isolates, and Implications for Integrase Inhibitors Efficacy. In, *International Journal of Molecular Sciences*. 2020.

Jazayeri, M.S., *et al.* Hepatitis B virus genotypes, core gene variability and ethnicity in the Pacific region. *J Hepatol* 2004;41(1):139-146.

Lin, S.Y.C., *et al.* Molecular Evolution and Phylodynamics of Acute Hepatitis B Virus in Japan. *PLOS ONE* 2016;11(6):e0157103.

Miura, T., *et al.* Genetic Characterization of Human Immunodeficiency Virus Type 1 in Elite Controllers: Lack of Gross Genetic Defects or Common Amino Acid Changes. *J Virol* 2008;82(17):8422-8430.
